# Assessment of direct and indirect associations between children active school travel and environmental, household and child factors using structural equation modelling

**DOI:** 10.1186/s12966-019-0794-5

**Published:** 2019-04-05

**Authors:** Erika Ikeda, Erica Hinckson, Karen Witten, Melody Smith

**Affiliations:** 1 School of Sport and Recreation, Auckland University of Technology, Auckland, 1647 New Zealand; 2 SHORE and Whariki Research Centre, Massey University, Auckland, 1010 New Zealand; 3 School of Nursing, The University of Auckland, Auckland, 1023 New Zealand

**Keywords:** Active travel, Independent mobility, Safety, Social environment, Built environment, Socio-ecological model

## Abstract

**Background:**

Active school travel (AST) is influenced by multiple factors including built and social environments, households and individual variables. A holistic theory such as Mitra’s Behavioural Model of School Transportation (BMST) is vital to comprehensively understand these complex interrelationships. This study aimed to assess direct and indirect associations between children’s AST and environmental, household and child factors based on the BMST using structural equation modelling (SEM).

**Methods:**

Data were drawn from Neighbourhoods for Active Kids (NfAK), a cross-sectional study of 1102 children aged 8–13 years (school years 5–8) and their parents from nine intermediate and 10 primary schools in Auckland, New Zealand between February 2015 and December 2016. Data were collected using an online participatory mapping survey (softGIS) with children, a computer-assisted telephone interviewing survey (CATI) with parents, and ArcGIS for built environment attributes. Based on the BMST a conceptual model of children’s school travel behaviour was specified for SEM analyses (‘hypothesised SEM’), and model modification was made to improve the model (‘modified SEM’). SEM analyses using Mplus were performed to test the hypothesised/modified SEM and to assess direct and indirect relationships among variables.

**Results:**

The overall fit of the modified SEM was acceptable (*N* = 542; Root mean square error of approximation = 0.04, Comparative fit index = 0.94, Tucker-Lewis index = 0.92). AST was positively associated with child independent mobility, child-perceived neighbourhood safety, and parent-perceived importance of social interaction and neighbourhood social environment. Distance to school, and parental perceptions of convenience and concerns about traffic safety were negatively associated with AST. Parental fears of stranger danger were indirectly related to AST through those of traffic safety. Distance to school and child independent mobility mediated relationships between AST and child school year and sex.

**Conclusions:**

Increasing children’s AST requires action on multiple fronts including communities that support independent mobility by providing child friendly social and built environments, safety from traffic, and policies that promote local schools and safe vehicle-free zones around school.

**Electronic supplementary material:**

The online version of this article (10.1186/s12966-019-0794-5) contains supplementary material, which is available to authorized users.

## Background

Active travel (e.g., walking or cycling to destinations) can be a convenient and regular way for children to accumulate physical activity. Children’s physical, psychological and social health and cognitive development benefit from active travel through opportunities to accumulate physical activity, interact with friends and nature, and spatially navigate their neighbourhood [1–3]. In more broad terms, active travel can also be economically beneficial and contribute to environmental sustainability via reducing traffic congestion and emissions due to motorised transportation.

There is a clear need for reducing the use of motorised transportation in favour of active travel. The school trip is one area where such changes could be achieved. In New Zealand (NZ) a majority of schools have zoning regulations, providing children who live inside the zone an absolute right to enrol at the school [4–7]. This means that many children might live close enough to the school they attend to actively travel to/from school (AST). Yet, recent data show less than half of NZ children aged 5–14 years get to school actively [8]. Demographic differences were also observed, with older youth (ages 10–14 years) and males more likely to report AST [8].

A wealth of studies have collectively demonstrated the complex nature of children’s AST [3, 9–13]. The diverse range of factors that can promote or inhibit children’s AST includes built and social environment factors as well as household and individual child factors. For the most part, these factors have been assessed using objective (e.g., geographic information systems (GIS)) and/or subjective (e.g., survey) measures [3, 9, 10, 13, 14]. The socio-ecological model has been the most commonly used to structure multiple layers of influence on AST [15–19].

A conceptual model specifically for children’s school travel behaviour, the Behavioural Model of School Transportation (BMST) was developed by Mitra [16]. The BMST is a comprehensive conceptual model that combines the socio-ecological model, a household active-travel approach [20] and McMillan’s framework [18] in which school travel behaviour is conceptualised as having two components: travel mode and accompaniment (i.e., independent versus escorted) [16]. Mitra identified four domains (external influences, urban environment, household, child) and five mediators (proximity to school, street connectivity, comfort and attractiveness of the travel route, traffic and personal safety, social capital) that influence children’s school travel behaviour. Previous studies have empirically tested the BMST; however, they were unable to examine indirect (mediated) relationships to AST [21] or missed integrating the social environment and children’s perceptions [22].

The application of theories such as BMST can highlight the structure of mediated relationships between variables such as built environments and safety [16, 18, 23]. Opportunities exist to improve the knowledge base through robust application of conceptual models to guide analytical techniques [11]. Given the complicated interrelationships of influences on AST, structural equation modelling (SEM) is an appropriate multivariate technique for testing theories and elucidating respective dependent relationships. The strength of SEM is the ability to combine analyses of linear and logistic regressions including direct and indirect (i.e., mediating) effects among observed and latent (i.e., unobserved) variables.

Yu and Zhu [24, 25] utilised SEM to evaluate two conceptual models for children’s walking to/from school. The first consisted of personal (including residential self-selection), social factors, and built environment factors (as a mediator of residential self-selection) [24]. The second considered personal, social and built environment factors and parental attitudes (as a mediator) [25]. Both models had acceptable/adequate fits. Children’s walking to/from school was negatively associated with attitudinal and walking barriers (e.g., too much to carry, too hot and sweaty) and safety concerns, and positively correlated with perceived proximity to school, enjoyment of walking and residential self-selection [24, 25]. These studies, however, did not incorporate objective built environment measures or children’s perspectives. Lu et al. [11] examined associations between children’s AST and child and parent self-efficacy using SEM based on Bandura’s social cognitive theory. This study examined relationships between children’s AST and psychological (i.e., self-efficacy), social and environmental factors. However, unlike Mitra’s BMST or other socio-ecological models [15–19], indirect relationships among these factors were not explicitly demonstrated. Mehdizadeh et al. [26] developed a more comprehensive conceptual model based on the social cognitive theory, the theory of planned behaviour and the prototype willingness model in which direct and indirect associations between children’s AST and built environment attributes, sociodemographic characteristics, as well as parent attitudes were conceptualised and tested using SEM. This model, however, did not integrate children’s perspectives.

**Fig. 1:**
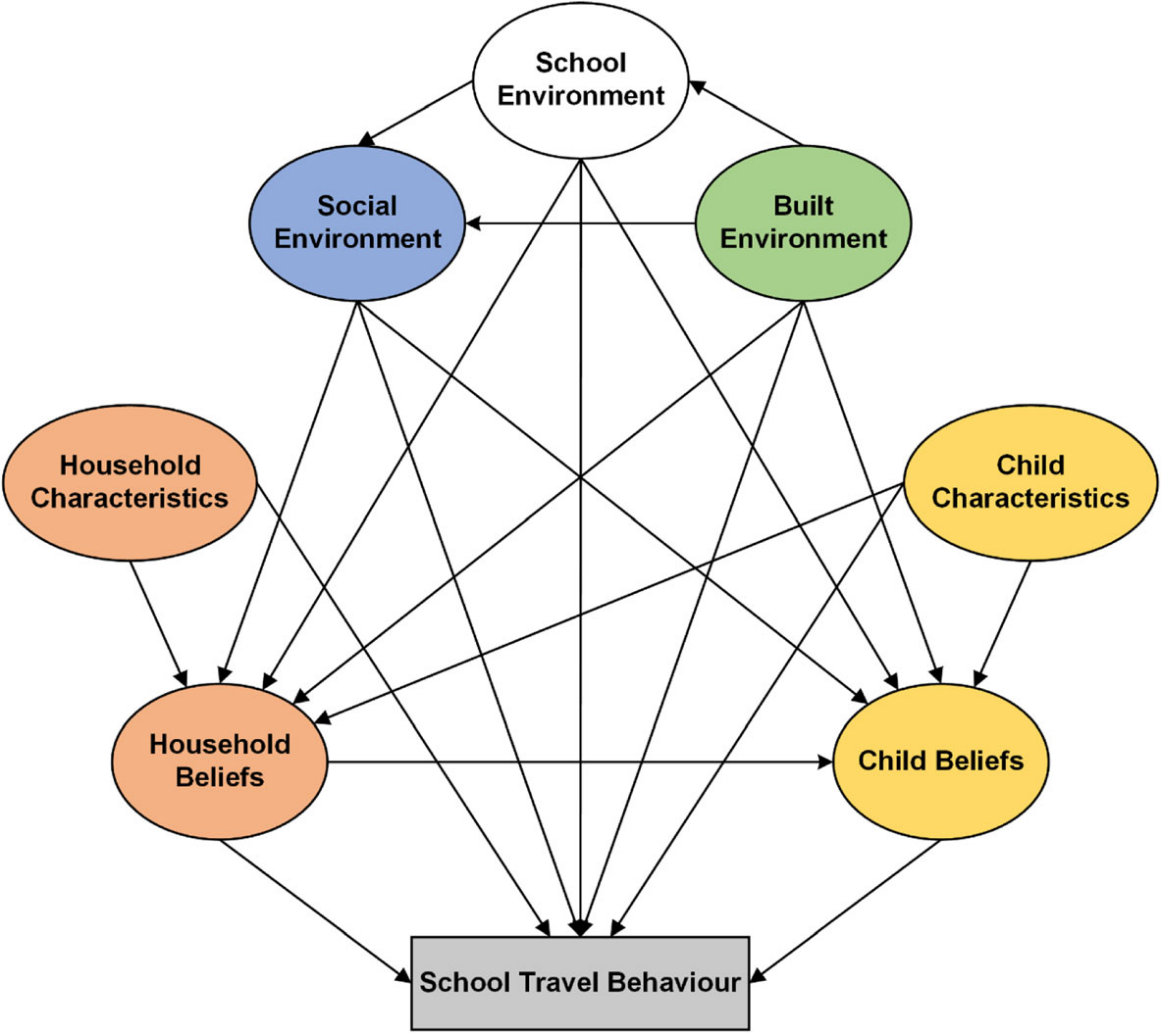
Children’s School Travel Behaviour Model (C-STBM)

The purpose of this paper is: (1) to develop and test a new model for use in children’s school travel behaviour, and (2) to assess direct and indirect associations between children’s AST and environmental, household and child factors based on Mitra’s BMST [16] using SEM. It is informed by a conceptual model developed from the BMST and the conceptual models designed by McMillan [18] and Panter et al. [19], entitled the Children’s School Travel Behaviour Model (C-STBM), as presented in Fig. 1. Six of the seven domains identified in the model (built environment, social environment, household characteristics, household beliefs, child characteristics, and child beliefs) were included in the current analysis. The seventh, the school environment (i.e., school policies, AST programmes) was not included due to an inadequate number of participating schools (see the section of ‘strengths and limitations’). The novelty of this study includes the simultaneous consideration of multiple factors across the socio-ecological spectrum, and inclusion of perceived/subjective and objectively assessed variables in relation to each other and to AST. We hypothesised that (1) the built environment, the social environment, household and child characteristics, and household and child beliefs were directly associated with children’s AST (Additional file 1); and (2) all the domains except child beliefs were indirectly related to children’s AST (Additional file 2).

## Methods

### Study design, setting, participants and protocol

New Zealand is characterised as a highly suburbanised nation with a total population of 4.9 million in 2018 of which 13% were children aged 5–14 years [27]. Auckland is NZ’s largest urban area (1.7 million in 2017) located in the North Island where the population has sprawled and shifted towards automobile dependency due to urban developments [28].

Neighbourhoods for Active Kids (NfAK) is a cross-sectional study conducted in Auckland that uses a child-centred approach to measuring and describing relationships between the built environment and a range of children’s activity behaviours and health outcomes. Information was collected using an online participatory mapping survey (i.e., softGIS) with children, a computer-assisted telephone interview (i.e., CATI) with parents, and geographic information systems (GIS) for built environment attributes. Design and methods of the full study are described in detail elsewhere [29].

Briefly, children aged 8–13 years (school years 5–8) and their parents from nine intermediate (middle/junior high) and 10 contributing primary (elementary) schools across nine neighbourhoods in Auckland, NZ participated in the study between February 2015 and December 2016. Schools were selected based on a matrix of school decile (i.e., a neighbourhood-level measure of socioeconomic status; high, medium, low), child-specific school walkability (high, medium, low) [30] and child-specific neighbourhood destination accessibility (NDAI-C; high, medium, low) [31]. This recruitment approach was applied to increase heterogeneity in neighbourhood deprivation and geographic characteristics.

A softGIS survey (https://maptionnaire.com) [32–34] was used to measure children’s mode of travel and route to school, perceived neighbourhood and traffic safety, and independent mobility. The software can be used on multiple platforms (i.e., tablet, computer), and the interface is similar to Google Maps but with functionality to add survey questions, marking of destinations, and the capture of location-specific information (e.g., likes/dislikes). The softGIS methodology allows participants to map their environment and social experiences at specific locations, as well as routes to destinations (e.g., from home to school) [32, 34]. Trained researchers visited schools during school hours at which time children completed a softGIS survey with one-on-one researcher support. Children were then asked to wear Actigraph GT3X+ accelerometers (Actigraph, Pensacola, FL) around their waist over seven consecutive days. A CATI survey was conducted with parents/caregivers of participating children to measure household sociodemographics, and reasons for decision-making on children’s school travel mode and relative importance of the reasons. Ethical approval to conduct the study was granted by the host institution ethics committees (AUTEC, 14/263, 3 September 2014; MUHECN 3 September 2014; UAHPEC 9 September 2014). Participant information sheets, child assent forms, and parent consent forms were provided to children. The children were asked to return their signed assent and parent consent forms within 2 weeks if they agreed to participate in the study.

### Measures

Information about observed variables including description of variables, type of variables (i.e., continuous, binary, ordinal, nominal), code or scale of variables, and descriptive statistics is summarised in Additional file 3.

#### School travel mode

Children’s usual mode of travel to school was self-reported using softGIS by asking “*How do you usually get to school?*” with responses being ‘walk’, ‘bike’, ‘scooter (non-motorised)’, ‘public bus, train or ferry’, ‘car, motorbike, scooter or taxi’, and ‘another way (e.g., skateboard)’. School travel mode was dichotomised to active travel (i.e., walk, bike, scooter, skateboard) and passive travel (i.e., car, public transport). Public transport (including school bus) was considered passive travel in this study. While public transport involves both active and passive travel modes (and so is associated with higher levels of physical activity than private motorised modes [35–37]), it was hypothesised that children spend more time in physically inactive behaviour (e.g., sitting) than active travel behaviour (e.g., walking from home to a bus stop or from a bus stop to school) for the school journey. Furthermore, school routes and their characteristics may be more similar between car and bus travel than between bus and active travel modes.

#### Child characteristics

Child’s school year (grade), sex and ethnicity were reported by schools or their parents/caregivers, and included in analyses as covariates. School-travel-related physical activity was assessed using Actigraph GT3X+ accelerometers (Actigraph, Pensacola, FL) during the 8:00 am-9:00 am commuting period on weekdays (Monday-Friday, excluding public holidays) [38]. Raw data were collected at frequency of 30 Hz, and aggregated to a 30 s epoch using Actilife v6 (Actigraph, Pensacola, FL) [39]. Accelerometer cut-points (vertical counts/min) provided by Evenson et al. [40] were utilised to classify time spent sedentary and in light, moderate and vigorous physical activity. Non-wear time was classified as 60 min or more of consecutive zeros counts [41]. Inclusion in analyses was a two-stage process. First, participants were required to have at least three valid days with a minimum of seven hours of wear time [42]. Of these, participants with at least two valid weekdays with 60 min of data between 8:00 am-9:00 am were included. The percentage of time spent (in minutes) in overall (i.e., light + moderate + vigorous) physical activity (PA) during the morning commute was calculated as:

$$ Physical\ activity=\left(\sum morning\ overall\  PA\div \sum allday\ overall\  PA\right)\times 100 $$

#### Child beliefs

Traffic safety perception was measured by the summed score of two items with a 4-point Likert scale (Spearman’s *ρ* = 0.29, *p* < 0.001) [43, 44]. Neighbourhood safety perception was measured by the summed score of two items with a 4-point Likert scale after combining responses of ‘hardly ever/never’ and ‘do not go out with/without an adult in the neighbourhood’ (*ρ* = 0.18, *p* < 0.001) [43, 44]. Independent mobility (i.e., unaccompanied/unsupervised travel) was assessed by the summed score of three items with a dichotomous response indicating whether the child had independent mobility or not (Cronbach’s *α* = 0.85) [44, 45].

#### Household characteristics

Parents/caregivers reported their highest academic qualification, their current employment situation, and number of adults, children aged under 18 years and working cars in their household.

#### Household beliefs

Importance of parent reasons for decision-making on children’s school travel mode was assessed by two items: “*What are the main reasons your child gets to school by respective school travel mode?*”, and “*How important would you say this reason when deciding how your child gets to school?*” Reasons were categorised into ‘distance to school’, ‘traffic safety’, ‘stranger danger’, ‘convenience’ and ‘social interaction’. Each reason was first dummy coded as ‘not main reason’ and ‘main reason’. ‘Main reason’ was then rated as ‘not important’, ‘a little bit important’, ‘important’, or ‘very important’.

#### Social environment

*Neighbourhood Social Environment* was a first-order factor (latent variable) which was collectively measured by three observed variables: neighbourhood safety, neighbourhood cohesion and neighbourhood connection [46]. The summed score was used to calculate factor scores of neighbourhood safety with nine items (Cronbach’s *α* = 0.76), neighbourhood cohesion with nine items (Cronbach’s *α* = 0.80), and neighbourhood connection with five items (Cronbach’s *α* = 0.85) [44]. A 5-point Likert scale was used, and scales were reverse coded where appropriate.

#### Built environment

SoftGIS home location (point) and child-drawn school route (polyline) data were downloaded from the softGIS survey, and imported into ArcGIS 10.2 (Environmental Systems Research Institute (ESRI), Redlands, CA). SoftGIS routes inside the school polygon were trimmed. All softGIS routes were manually cleaned and obviously incorrect softGIS routes (e.g., incomplete routes, routes ended at non-school locations) were excluded from further analyses. Distance to school (in metres) along softGIS routes was calculated, and log-transformed. SoftGIS routes were then buffered using a 80 m radius on each side of the street centre line to measure built environment attributes [47].

*Active Mobility Environment* was a first-order factor (latent variable) which was collectively assessed by four observed variables: residential density, street connectivity, high traffic exposure and low traffic exposure. Residential density was calculated as the ratio of residential dwellings to the residential land area (i.e., without water) of 80 m softGIS route buffer [47]. Meshblock level data on the number of private occupied dwellings at the 2013 Census was downloaded from the Statistics New Zealand and linked to the meshblock boundaries. Street connectivity was calculated as the ratio of number of intersections with three or more intersecting streets to the land area of 80 m softGIS route buffer [47]. Road centreline data were obtained from the 2015 CoreLogic Transport dataset. High or low traffic exposure was measured by length of high or low traffic roads within a 80 m softGIS route buffer weighted by an inverse softGIS route distance:

$$ High\ (Low)\  traffic\ exposure=\frac{1}{\delta r\div {\sum}_r\delta r\times {10}^6}\times length\ of\ high\ (low)\  traffic\ r oads $$

where *δr* is the distance of an individual softGIS route, ∑_*r*_*δr* is the sum of softGIS route distances (i.e., a shorter softGIS route distance had a higher weight) [48]. Road classification derived from the 2015 CoreLogic Transport dataset was employed as a proxy for traffic volume [47].

### Statistical analysis

#### Structural equation modelling

Structural equation modelling using Mplus version 8.1 [49] was employed to test the hypothesised conceptual model (Additional file 4). SEM is a multivariate technique combining factor analysis and multiple regression, which can encompass two components: a measurement model (i.e., confirmatory factor analysis) and a structural model [50, 51]. Benefits of SEM are (1) to represent theoretical concepts which cannot be directly observed, (2) to improve the statistical estimation of relationships between the concepts by considering the measurement error, (3) to estimate multiple and interrelated dependent relationships, and (4) to define a model to elucidate the complete set of relationships between variables [51].

Mplus can estimate mixture modelling with cross-sectional data including combinations of continuous, binary, ordinal, and nominal observed variables, and can handle missing data [49]. Multiple imputation using Bayesian analysis was performed for a set of observed variables with missing values (100 replications) [49]. As the children were nested within their schools, the data might have a multilevel hierarchical structure (i.e., a multilevel model) [52]. Intraclass correlation coefficients (ICCs) were performed to examine the clustered data structure (i.e., the variability in observed variables can be explained by schools). The ICCs indicated cluster effects might exist in AST (ICC = 0.13), year (ICC = 0.81), ethnicity (NZ European: ICC = 0.39, Pacific: ICC = 0.30), independent mobility (ICC = 0.22), education (ICC = 0.15), neighbourhood safety (ICC = 0.19), and GIS measures (ICC = 0.15–0.44). However, due to the small size of school clusters (*N* = 19), a multilevel model was deemed inappropriate.

A measurement model specified observed variables for each latent variable (i.e., *Active Mobility Environment* and *Neighbourhood Social Environment*). The construct validity including convergent validity, discriminant validity and reliability of the measurement model was assessed. Convergent validity was assessed using factor loadings (λ; ≥0.5), average variance extracted (AVE; ≥0.5), and construct reliability (CR; ≥0.7). Discriminant validity was assessed by a correlation between the latent variables being significantly smaller than 1.0.

A structural model was specified based on the hypothesised conceptual model by assigning direct and indirect (mediating) dependent relationships to AST (Additional files 1 and 2). The specific indirect (mediating) effect represents a pathway from an independent variable to AST through a mediator and can be classified as full (100% mediation and no direct effects on AST) or partial (some mediation and some remaining direct effect on AST) [53]. Individual estimates of each hypothesised structural relationship were examined by the significance (i.e., *p* < 0.05, *p* < 0.01) and direction (i.e., positive, negative; Additional file 1) of the standardised associations.

#### Modelling strategy

A model development strategy was applied to improve the conceptual model of children’s school travel behaviour. Two stages were involved: (1) testing the hypothesised SEM, and (2) developing the SEM through modifications of the measurement or structural models [51]. The SEM developed through the second stage should be tested with an independent sample from the first stage [51]. Therefore, the current sample (*N* = 1085) was randomly divided into two groups (Stage 1: *N* = 543 and Stage 2: *N* = 542). Chi-square tests and t-tests were conducted using IBM SPSS Statistics v24 (IBM Cooperation, USA) to test for differences in observed variables between the two groups. No significant differences were observed between the Stage 1 and Stage 2 groups.

#### Estimation and goodness-of-fit

The weighted least squares means and variance adjusted (WLSMV) estimation was used for analysis of categorical outcomes (e.g., school travel mode) [49, 54]. To assess how well the specified model reproduced the observed covariance matrix, four (two absolute and two incremental) fit indices were employed: standardised root mean residual (SRMR), root mean square error of approximation (RMSEA), comparative fit index (CFI) and Tucker-Lewis index (TLI) [50, 51, 55, 56]. The two-index presentation strategy with at least one absolute (e.g., SRMR, RMSEA) and one incremental (e.g., CFI, TLI) fit indices were recommended [51, 56]. The SRMR was only reported for the measurement model because Mplus did not produce the SRMR for binary outcomes in the structural model where the RMSEA was reported [52]. Cut-off criteria for a ‘good’ fit were defined as SRMR≤0.08, RMSEA≤0.06, CFI ≥ 0.95 and TLI ≥ 0.95 [56]. SRMR≤0.08, RMSEA≤0.08, CFI ≥ 0.90 and TLI ≥ 0.90 were considered as an ‘acceptable/adequate’ fit [51, 57].

## Results

**Fig. 2:**
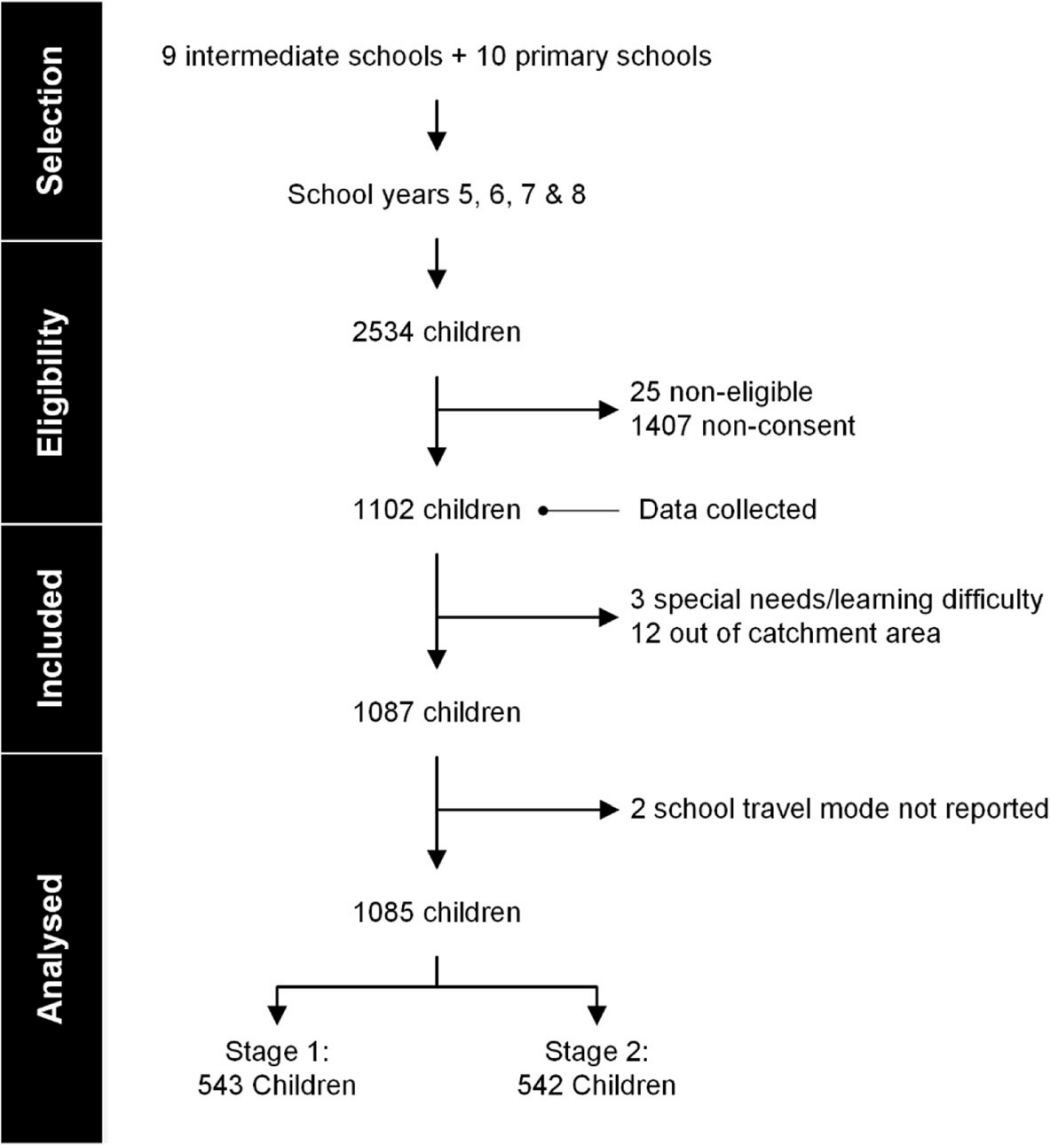
Flow of recruitment and data analyses

Figure 2 presents a flow chart of children recruited into the current study and those retained in analyses. Seventeen out of the 1102 study participants were excluded due to having special needs or a learning difficulty (*N* = 3), living out of the school catchment zone (*N* = 12), or having missing data for school travel mode (*N* = 2). Data from 1085 participants were included in analyses. Descriptive statistics for observed variables are presented in Table 1 and Additional file 3.

**Table 1:** Information about observed variables and their descriptive statistics (*N* = 1085)

Observed variable	Latent variable	Description	Data source	Measurement scale	Descriptive statistics†
School Travel Mode					
Active school travel	–	How do you usually get to school?	SoftGIS	Passive travel:	58.0%
				Car	46.1%
				Public transport	11.9%
				Active travel:	42.0%
				Walk	34.4%
				Bike	3.9%
				Scooter, skateboard	3.8%
Child Characteristics					
Year	–	Child’s school year	School/Parent consent form	Year 5	24.5%
				Year 6	26.4%
				Year 7	24.2%
				Year 8	24.9%
Sex	–	Child’s sex	School/Parent consent form	Male	49.0%
				Female	51.0%
Ethnicity	–	Child’s ethnicity	School/CATI	New Zealand (NZ) European	52.7%
				Māori	12.9%
				Pacific	15.3%
				Asian	15.0%
Physical activity	–	Percentage of time spent in overall (light + moderate + vigorous) physical activity during the morning (8:00–9:00 am) commute	Accelerometer	–	8.8 ± 3.0
Child Beliefs					
Traffic safety	–	1. The roads around my school are busy with traffic before and after school.	SoftGIS	All of the time / Most of the time	53.7%
				Sometimes / Hardly ever / Never	46.0%
		2. The roads around my school are full of parked cars before and after school.	SoftGIS	All of the time / Most of the time	53.3%
				Sometimes / Hardly ever / Never	46.2%
Neighbourhood safety	–	1. If I am out with an adult, I feel safe in my neighbourhood.	SoftGIS	Sometimes / Hardly ever / Never/ Do not go out with an adult in the neighbourhood	10.0%
				All of the time / Most of the time	89.6%
		2. If I go out without an adult, I feel safe in my neighbourhood.	SoftGIS	Sometimes / Hardly ever / Never / Do not go out without an adult in the neighbourhood	45.2%
				All of the time / Most of the time	54.6%
Independent mobility	–	1. Are you allowed to cross main roads on your own?	SoftGIS	No	32.3%
				Yes	67.4%
		2. Are you allowed to go on local buses or trains or ferries on your own?	SoftGIS	No	71.2%
				Yes	27.9%
		3. If you have a bicycle, are you allowed to ride it to go to places?	SoftGIS	No / Do not have a bicycle	40.5%
				Yes	58.9%
Household Characteristics					
Education	–	What is your highest academic qualification?	CATI	Certificate (levels 1–6), Diploma or lower	51.2%
				Bachelor’s degree or higher	30.0%
Employment	–	Which one best describes your main current employment situation?	CATI	Full-time	40.0%
				Part-time	25.0%
Number of adults	–	How many adults, including yourself, live in your household?	CATI	1–2 adults	65.6%
				Greater than or equal to 3 adults	16.2%
Number of children	–	How many other children under 18 live in your household?	CATI	No other children	12.1%
				1–2 children	58.0%
				Greater than or equal to 3 children	11.7%
Car ownership	–	How many working cars are available to your household?	CATI	Less than or equal to 1 car	18.1%
				Greater than or equal to 2 cars	63.8%
Household Beliefs					
Distance to school	–	What are the main reasons your child gets to school by (travel mode to school)? How important would you say this reason (i.e., distance to school) when deciding how your child gets to school?	CATI	Not main reason	35.0%
				Not / A little bit important	1.4%
				Important / Very important	41.9%
Traffic safety	–	What are the main reasons your child gets to school by (travel mode to school)? How important would you say this reason (i.e., traffic safety) when deciding how your child gets to school?	CATI	Not main reason	78.5%
				Not / A little bit important	0.2%
				Important / Very important	6.3%
Stranger danger	–	What are the main reasons your child gets to school by (travel mode to school)? How important would you say this reason (i.e., stranger danger) when deciding how your child gets to school?	CATI	Not main reason	79.6%
				Not / A little bit important	0.2%
				Important / Very important	5.6%
Convenience	–	What are the main reasons your child gets to school by (travel mode to school)? How important would you say this reason (i.e., convenience) when deciding how your child gets to school?	CATI	Not main reason	56.0%
				Not / A little bit important	3.4%
				Important / Very important	23.3%
Social interaction	–	What are the main reasons your child gets to school by (travel mode to school)? How important would you say this reason (i.e., social interaction) when deciding how your child gets to school?	CATI	Not main reason	80.8%
				Not / A little bit important	0.7%
				Important / Very important	4.2%
Social environment					
Neighbourhood safety	Neighbourhood social environment	1. There are safe places for children to play in our neighbourhood.	CATI	Strongly disagree / Disagree	12.7%
				Neither agree nor disagree	5.9%
				Agree / Strongly agree	62.2%
		2. It’s a good place to bring up children.	CATI	Strongly disagree / Disagree	3.3%
				Neither agree nor disagree	5.1%
				Agree / Strongly agree	72.9%
		3. I feel safe walking down my street after dark.	CATI	Strongly disagree / Disagree	22.0%
				Neither agree nor disagree	7.0%
				Agree / Strongly agree	51.5%
		4. I worry about the number of crimes committed in our neighbourhood.	CATI	Strongly disagree / Disagree	38.2%
				Neither agree nor disagree	11.2%
				Agree / Strongly agree	31.4%
		5. Graffiti and vandalism are problems.	CATI	Strongly disagree / Disagree	58.8%
				Neither agree nor disagree	5.3%
				Agree / Strongly agree	17.3%
		6. Roaming dogs are a problem in our neighbourhood.	CATI	Strongly disagree / Disagree	62.3%
				Neither agree nor disagree	4.1%
				Agree / Strongly agree	14.8%
		7. It’s a good place to buy a home.	CATI	Strongly disagree / Disagree	6.7%
				Neither agree nor disagree	3.5%
				Agree / Strongly agree	70.3%
		8. Bullying is a problem in our neighbourhood.	CATI	Strongly disagree / Disagree	10.8%
				Neither agree nor disagree	5.6%
				Agree / Strongly agree	60.9%
		9. There are a lot of families with young children living in our neighbourhood.	CATI	Strongly disagree / Disagree	6.1%
				Neither agree nor disagree	4.5%
				Agree / Strongly agree	69.0%
Neighbourhood cohesion	Neighbourhood social environment	1. People are willing to help.	CATI	Strongly disagree / Disagree	5.5%
				Neither agree nor disagree	7.7%
				Agree / Strongly agree	64.5%
		2. Neighbours watch out for kids.	CATI	Strongly disagree / Disagree	7.0%
				Neither agree nor disagree	7.4%
				Agree / Strongly agree	62.6%
		3. It’s a close knit neighbourhood.	CATI	Strongly disagree / Disagree	19.4%
				Neither agree nor disagree	15.3%
				Agree / Strongly agree	44.5%
		4. I could borrow $10 from a neighbour.	CATI	Strongly disagree / Disagree	22.7%
				Neither agree nor disagree	5.1%
				Agree / Strongly agree	46.9%
		5. If there is a problem with neighbours, we can deal with it.	CATI	Strongly disagree / Disagree	5.4%
				Neither agree nor disagree	5.1%
				Agree / Strongly agree	67.9%
		6. The neighbours cannot be trusted.	CATI	Strongly disagree / Disagree	65.4%
				Neither agree nor disagree	6.0%
				Agree / Strongly agree	7.0%
		7. People will take advantage of you.	CATI	Strongly disagree / Disagree	64.6%
				Neither agree nor disagree	5.3%
				Agree / Strongly agree	7.6%
		8. People you don’t know will greet you or say hello to you.	CATI	Strongly disagree / Disagree	6.9%
				Neither agree nor disagree	5.9%
				Agree / Strongly agree	68.0%
		9. People of different backgrounds don’t talk to each other.	CATI	Strongly disagree / Disagree	52.9%
				Neither agree nor disagree	7.6%
				Agree / Strongly agree	17.9%
Neighbourhood connection	Neighbourhood social environment	1. Parents in this neighbourhood know their children’s friends.	CATI	Strongly disagree / Disagree	8.0%
				Neither agree nor disagree	6.1%
				Agree / Strongly agree	61.1%
		2. Adults in this neighbourhood know who the local children are.	CATI	Strongly disagree / Disagree	11.0%
				Neither agree nor disagree	10.6%
				Agree / Strongly agree	52.8%
		3. There are adults in this neighbourhood that the children can look up to.	CATI	Strongly disagree / Disagree	9.8%
				Neither agree nor disagree	10.0%
				Agree / Strongly agree	51.9%
		4. Parents in this neighbourhood generally know each other.	CATI	Strongly disagree / Disagree	13.0%
				Neither agree nor disagree	10.9%
				Agree / Strongly agree	53.5%
		5. You can count on adults in this neighbourhood to watch out that children are safe and don’t get in trouble.	CATI	Strongly disagree / Disagree	8.6%
				Neither agree nor disagree	10.2%
				Agree / Strongly agree	56.3%
Built environment					
Distance to school	–	Distance to school (in metres) along softGIS school routes	GIS	–	2783.7 ± 3557.7
		Distance to school (log-transformed) along softGIS school routes	GIS	–	7.4 ± 1.0
Residential density	Active mobility environment	Ratio of residential dwellings to the residential land area (i.e., without water) of 80 m softGIS route buffer	GIS	–	28.8 ± 10.8
Street connectivity	Active mobility environment	Ratio of number of intersections with three or more intersecting streets to the land area of 80 m softGIS route buffer	GIS	–	56.6 ± 19.2
High traffic exposure	Active mobility environment	Length of high traffic roads within 80 m softGIS route buffer weighted by inverse softGIS route distance	GIS	–	5.9 ± 4.9
Low traffic exposure	Active mobility environment	Length of low traffic roads within 80 m softGIS route buffer weighted by inverse softGIS route distance	GIS	–	10.5 ± 8.2

CATI = computer-assisted telephone interviewing. GIS = geographic information systems. †Frequencies (%) for binary or ordinal variables; mean ± standard deviation for continuous variables

### Model modification

A full SEM including the measurement and structural models are illustrated in Additional file 4. The hypothesised SEM produced unacceptable/inadequate fit indices with RMSEA = 0.07, CFI = 0.64 and TLI = 0.55 (Stage 1: *N* = 543). Linear and logistic regressions were conducted to identify non-significant observed variables associated with AST or the other observed variables [25]. The results of regressions and theoretical evidence were considered to modify the hypothesised SEM. Parents’ highest academic qualification (i.e., education), their employment status (i.e., full-time, part-time), number of adults in their household, and associated dependent relationships with these observed variables were removed through the modification process. A majority of the interviewees for the CATI were mothers of the child (69.2%) followed by fathers of the child (14.2%), suggesting results of parent education and employment may have been biased towards those of mothers.

### Measurement model

*Active Mobility Environment* was specified by a combination of exploratory factor analysis and theory, comprising four observed variables: residential density, street connectivity, high traffic exposure and low traffic exposure (Additional file 1). *Neighbourhood Social Environment* was specified based on theory [46], encompassing three observed variables: neighbourhood safety, neighbourhood cohesion and neighbourhood connection (Additional file 1). Fit indices showed that the measurement model was acceptable with SRMR = 0.06, CFI = 0.93 and TLI = 0.91 (Stage 2: *N* = 542). Results of the construct validity of the measurement model denoted good validity and reliability with standardised factor loadings (λ) ranging from 0.50 to 1.00; AVEs of 0.59 (*Active Mobility Environment*) and 0.62 (*Neighbourhood Social Environment*); and CRs of 0.84 (*Active Mobility Environment*) and 0.82 (*Neighbourhood Social Environment*). A correlation between *Active Mobility Environment* and *Neighbourhood Social Environment* was significantly smaller than 1.0 (95% confidence interval: − 0.17-0.05), indicating good discriminant validity.

### Structural model

**Fig. 3:**
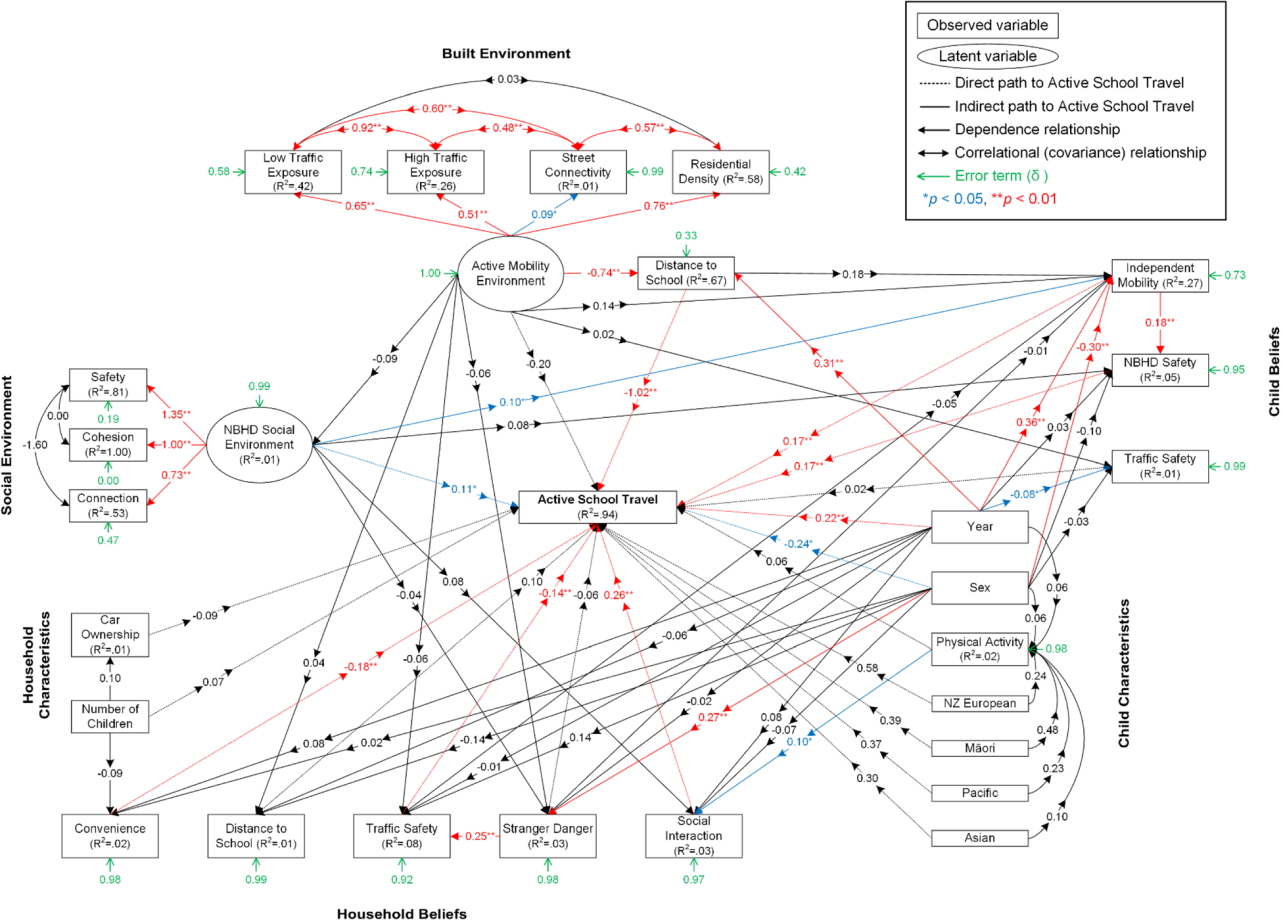
Standardised estimated coefficients of the structural equation model of children’s active travel to school. Root mean square error of approximation (RMSEA) = 0.04, comparative fit index (CFI) = 0.94, Tucker-Lewis index (TLI) = 0.92

The overall fit of the modified SEM was acceptable/adequate with RMSEA = 0.04, CFI = 0.94 and TLI = 0.92 (Stage 2: *N* = 542). The modified SEM accounted for 94.4% of the variance in AST. Standardised and unstandardised relationships between the observed/latent variables and AST are presented in Fig. 3 and Additional file 5.

#### Direct effects

Children in higher school year (estimate = 0.22, *p* < 0.01) and more males than females (estimate = − 0.24, *p* < 0.05) were more likely to actively travel to school. Neighbourhood safety (estimate = 0.17, *p* < 0.01), independent mobility (estimate = 0.17, *p* < 0.01), importance of social interaction (estimate = 0.26, *p* < 0.01), and *Neighbourhood Social Environment* (estimate = 0.11, *p* < 0.05) were significantly and positively associated with AST. Significantly negative associations with AST were found for importance of traffic safety (estimate = − 0.14, *p* < 0.01), importance of convenience (estimate = − 0.18, *p* < 0.01), and distance to school (estimate = − 1.02, *p* < 0.01). Distance to school had the strongest direct association with AST among the observed and latent variables.

#### Specific indirect (mediating) effects

**Fig. 4:**
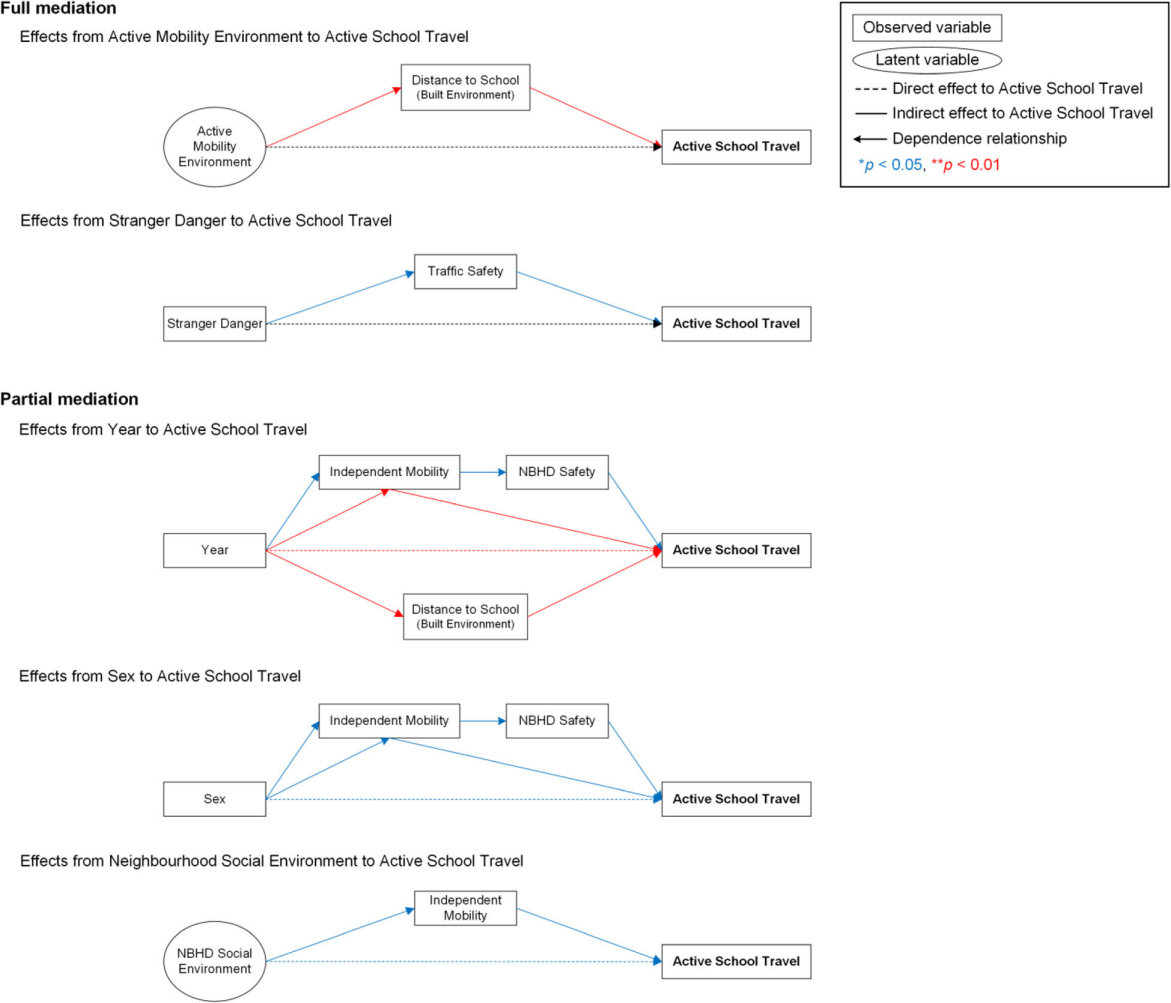
Standardised specific indirect effects on children’s active travel to school

Specific indirect (mediating) effects from the observed/latent variables to AST are shown in Fig. 4. A full mediation was observed in the pathway from *Active Mobility Environment* to AST through distance to school (*p* < 0.01). All indicators of *Active Mobility Environment* (i.e., residential density, street connectivity, high and low traffic exposure) were negatively correlated with distance to school (*r* = − 0.61, − 0.06, − 0.42 and − 0.52, respectively; standard errors for the correlation matrix were not available in Mplus). The pathway from importance of stranger danger to AST was fully mediated by importance of traffic safety (*p* < 0.05).

Independent mobility partially mediated the pathways from school year (*p* < 0.01), sex (*p* < 0.05) and *Neighbourhood Social Environment* (*p* < 0.05) to AST. The pathways from school year (*p* < 0.05) or sex (*p* < 0.05) to AST were also partially mediated by independent mobility through neighbourhood safety. Distance to school was a partial mediator of the pathway from school year to AST (*p* < 0.01).

## Discussion

This study developed and tested a new model (i.e., C-STBM) for use in children’s school travel behaviour, in which the dynamic interrelationships between children’s AST and multiple environmental factors were conceptualised. Direct and indirect associations between children’s AST and the built environment, the social environment, household and child characteristics, and household and child beliefs were comprehensively assessed using SEM. The modified SEM demonstrated acceptable/adequate model fit, explaining 94.4% of the variance in AST. This study shows that children’s AST has a complex structure and demonstrates how multiple factors at the individual level were interrelated. Distance to school and independent mobility had a multifaceted function including making a direct impact on children’s AST but also acting as mediators influenced by child characteristics such as school year and sex. Older (i.e., intermediate school) and male children were more likely to actively travel to school than younger (i.e. primary school) and female children. Parental perceptions of convenience, traffic safety and social interactions as well as child perception of neighbourhood safety were mutually associated with children’s AST.

### Distance to school

In agreement with findings from previous studies, distance to school was strongly associated with AST and increased by school year [3, 9, 10, 16, 26, 58–61]. Our measure of *Active Mobility Environment* was not directly associated with AST but was mediated by distance to school, suggesting that urban environments that support active mobility (i.e., increased residential density and street connectivity as well as less busy roads) can shorten distance to school and encourage AST.

In NZ, parents tend to choose their children’s primary school based on the quality of school resources and the overall school reputation rather than the accessibility of school (e.g., within walking distance, accessible public transport) or living within the school zone (i.e., ‘reasonably convenient’ local schools) [6, 62]. This tendency continues into secondary schools where school zoning policies have been ‘guidelines’, and adolescents and/or parents have freedom of their school choice [63]. In fact, less than a third of adolescents chose their school because of proximity to school [63]. In this respect, future interventions should consider strategies for children living far from school to encourage them to incorporate active and passive travel rather than only passive travel (e.g., door-to-door chauffeuring). For example, a drop-off/pick-up zone can be arranged away from school entrances so that every child has an opportunity to walk to school within the ‘vehicle-free’ area. This approach can also ease traffic congestion at school and protect active travellers from traffic danger [64].

Public transport is underutilised in NZ for school travel. This study showed 11.2% of the primary and 45.7% of the intermediate children had parental permission to use public transport on their own. However, only 2.9% of the primary and 21.2% of the intermediate school children used public transport to school (cf. car = 56.0 and 35.8%, respectively). Building safe neighbourhoods and supporting parents using a step-by-step approach to improve children’s independent mobility can be practical future interventions [13, 65]. For instance, potential first steps could be arranging a drop-off point for walking school buses and ensuring safe places to cross in the immediate school vicinity.

### Convenience

Children were less likely to actively travel to school if their parents prioritised convenience as a reason for choosing their school travel modes. Research has shown that parents of children who use active travel modes and those who use passive travel modes can both perceive their school travel mode as convenient or easy [26, 66]. However, parents of passive travellers more often quoted its convenience or ease in terms of their time, distance and schedules [60, 66–68]. In addition, trip chaining by car has been viewed as the best and least stressful way for working parents and/or parents who have more than one child in their household to move around multiple destinations including schools [66, 69–71]. Consistent with existing findings, stronger perceptions of convenience was associated with the use of passive travel modes. Paradoxically, if children travel to school independently, parents have less need to juggle their home and work schedules. In this regard, the notion of convenience may not be simply interpreted, and other reasons such as safety can be intermingled. School Travel Plans (e.g., walking school buses, cycle trains) programmes, for instance, can make AST safe, enjoyable and sociable for children [72], which may balance out parental perceptions of convenience to use cars.

### Independent mobility

Independent mobility was not only positively associated with AST, but also acted as a mediator between AST and school year, sex, neighbourhood safety and *Neighbourhood Social Environment*, suggesting the important role of independent mobility for AST in its own right. These findings supported empirical evidence from previous studies that independent mobility is influenced by child’s age, sex, and the quality of neighbourhood environments (e.g., traffic safety) [45, 46, 73–78]. Community engagement to create child-friendly and safe environments can allow children to be independent in their neighbourhood [79]. Social pressure and expectations of being a ‘good parent’ can make parents anxious about travel practices and the safety of their children [67]. Future research should identify parental concerns and investigate community strategies to increase social surveillance and ‘eyes’ for active travellers in the neighbourhood [65] to help reverse social expectations so that independent mobility becomes associated with ‘good parenting’. Policy support for such an approach is also needed [45]. For example, in NZ, parents are not allowed to leave their children under the age of 14 years without reasonable supervision and care [80] wherein the idea of independent mobility may be questioned by parents. Further, policy-makers and school communities would be wise to take children’s needs and views into account using a participatory process, and involve them in decision making and policy implementation.

### Safety

If parents reported that traffic safety and stranger danger were important for decision-making regarding their children’s school travel mode, children were less likely to actively travel to school. Parental perceptions of traffic safety (e.g., traffic accidents and congestion) and stranger danger (e.g., crime, kidnapping) have been recognised as key obstacles to AST [9, 13, 66, 81]. As Safe Routes to School programmes proved, traffic safety can be improved by providing walking and cycling infrastructure (e.g., sidewalks, speed bumps, crosswalks, cycle lanes, traffic signals) [82, 83]. Educational programmes including the development of motor and cognitive skills can be also effective to enhance children’s self-efficacy and parents’ confidence about their children’s abilities to actively travel to school under the traffic environment [16, 84]. Despite actual risks of stranger danger happening on rare occasions, the extreme cases were often exaggerated by the media; consequently, parental fear and anxiety of stranger danger were overly stressed [65, 79, 85].

### Social interaction and physical activity

The importance of social interaction was positively associated with AST and physical activity specifically during the morning commute (8:00 am-9:00 am). The findings demonstrated that the choice of AST viewed as an opportunity for social interactions can be coupled with a way to accumulate physical activity. Egli et al. [86] revealed that children enjoyed interacting with their friends and family on the route to school. Tarp et al. [87] reported, irrespective of bout-duration, time spent at higher intensity physical activity (i.e., 3000 counts per minutes, equivalent to walking speed at approximately 66–83 m/min) was inversely associated with cardiometabolic risk factors. In light of a child’s average walking speed of 65–83 m/min [88–90], arguably contributing to ‘light to moderate’ intensity activity, walking to school for 10 min can provide a great deal of health benefits and be an achievable goal and a practical intervention for children’s regular accumulation of physical activity.

### Strengths and limitations

This study used a SEM technique to comprehensively understand the complex interrelationships between children’s AST and environmental, household and child factors. To our knowledge, this is the first study to incorporate voices from children (softGIS) and parents (CATI) and objective GIS measures in a model which was a novel and holistic approach and advantageous to test and develop the conceptual model of children’s school travel behaviour. Use of child-drawn routes to school using softGIS to generate route environment measures in the current study is likely to have provided greater specificity of the built environment children actually encounter en-route to school compared to calculating these measures using the more common method of GIS-modelled shortest routes [47]. However, self-report bias might exist through participants’ recall, spatial knowledge and online map navigation skills, as well as cognitive abilities [91, 92].

The absence of school cluster analysis and unavailability of observed variables in analyses were limitations of this study. The use of a multilevel model is recommended for data structured by multiple levels (i.e., individuals and clusters/groups). The effect of clusters (i.e., schools) and group level (i.e., school environment) data such as school policies and AST programmes can influence children’s AST. Future research should consider a larger school sample size (at least *N* > 20 clusters, ideally *N* > 50) to perform multilevel analyses in SEM.

Though observed variables were cautiously formulated based on the conceptual model, some of the key observed variables were not accessible in this study. Examples include GIS measures of walking/cycling infrastructure [58, 70, 93, 94], household socioeconomic status [9, 13, 95], child/parent attitudes towards AST [96–98], and child self-efficacy [95, 99]. Finally, the causal interpretation of the findings cannot be obtained due to the cross-sectional study design. The findings are also applicable only in the context of the urbanised Auckland region in NZ, and may not be generalisable to different geographic locations.

## Conclusions

Increasing children’s AST requires action on multiple fronts including communities that support independent mobility by providing child friendly social and built environments, safety from traffic, and policies that promote local schools and safe vehicle-free zones around school.

## Additional file

Additional file 1: Hypothesised direct relationships between children’s school travel behaviour and the built environment, the social environment, household and child characteristics, and household and child beliefs. (DOCX 98.3 kb)

Additional file 2: Hypothesised indirect relationships between children’s school travel behaviour and the built environment, the social environment, household and child characteristics, and household and child beliefs. (DOCX 95.1 kb)

Additional file 3: **Table S1.** Information about observed variables and their descriptive statistics. (*N* = 1085) (DOCX 63.7 kb)

Additional file 4: The hypothesised full structural equation modelling. (DOCX 561 kb)

Additional file 5: Unstandardised estimated coefficients of the structural equation model of children’s active travel to school. Root mean square error of approximation (RMSEA) = 0.04, comparative fit index (CFI) = 0.94, Tucker-Lewis index (TLI) = 0.92. (DOCX 606 kb)

## Abbreviations

AST: Active school travel
AVE: Average variance extracted
BMST: Behavioural Model of School Transportation
CATI: Computer-assisted telephone interviewing survey
CFA: Confirmatory factor analysis
CFI: Comparative fit index
CR: Construct reliability
C-STBM: Children’s School Travel Behaviour Model
EFA: Exploratory factor analysis
GIS: Geographic information systems
ICC: Interclass correlation coefficient
MVPA: Moderate-to-vigorous intensity physical activity
NDAI-C: Child-specific neighbourhood destination accessibility
NfAK: Neighbourhoods for Active Kids
NZ: New Zealand
RMSEA: Root mean square error of approximation
SEM: Structural equation modelling
SRMR: Standardised root mean residual
TLI: Tucker-Lewis index
WLSMV: Weighted least squares means and variance adjusted
