# Supplementary material for: Assessment of direct and indirect associations between children active school travel and environmental, household and child factors using structural equation modelling
Source: Int J Behav Nutr Phys Act. 2019 Apr 5;16:32. doi: 10.1186/s12966-019-0794-5 (PMC6451289; doi:10.1186/s12966-019-0794-5)
Supplement: Supplementary file 1 — Hypothesised direct relationships between children’s school travel behaviour and the built environment, the social environment, household and child characteristics, and household and child beliefs. (DOCX 98.3 kb) [file 12966_2019_794_MOESM1_ESM.docx]

# Additional file 1

Hypothesised direct relationships between children’s school travel behaviour and the built environment, the social environment, household and child characteristics, and household and child beliefs

| **Domain** | **Observed variable** | ***Latent variable*** | **Association** | **References** |
| --- | --- | --- | --- | --- |
| Built Environment | Residential density | *Active mobility environment* | Positive | [[1-4](#_ENREF_1)] |
|  | Street connectivity | *Active mobility environment* | Positive | [[1-4](#_ENREF_1)] |
|  | High traffic exposure | *Active mobility environment* | Negative | [[1](#_ENREF_1), [5-7](#_ENREF_5)] |
|  | Low traffic exposure | *Active mobility environment* | Positive | [[1](#_ENREF_1), [5](#_ENREF_5)] |
|  | Distance to school | - | Negative | [[1-4](#_ENREF_1), [8-11](#_ENREF_8)] |
| Social Environment | Safety | *Neighbourhood social environment* | Positive | [[12-14](#_ENREF_12)] |
|  | Cohesion | *Neighbourhood social environment* | Positive | [[12](#_ENREF_12), [13](#_ENREF_13)] |
|  | Connection | *Neighbourhood social environment* | Positive | [[13](#_ENREF_13)] |
| Household Characteristics | Car ownership | - | Negative | [[1](#_ENREF_1), [3](#_ENREF_3), [6](#_ENREF_6), [11](#_ENREF_11), [14-17](#_ENREF_14)] |
|  | Number of children | - | Positive | [[1](#_ENREF_1), [2](#_ENREF_2), [12](#_ENREF_12), [16](#_ENREF_16), [18](#_ENREF_18)] |
|  | Number of adults* | - | Negative | [[1](#_ENREF_1), [16](#_ENREF_16)] |
|  | Parent education* | - | Negative | [[1](#_ENREF_1), [11](#_ENREF_11), [14](#_ENREF_14), [16](#_ENREF_16), [19](#_ENREF_19)] |
|  | Parent employment (reference category: unemployed)* | - | Negative | [[1](#_ENREF_1), [3](#_ENREF_3), [4](#_ENREF_4), [9](#_ENREF_9), [20](#_ENREF_20), [21](#_ENREF_21)] |
| Household Beliefs | Importance of social interaction | - | Positive | [[9](#_ENREF_9), [14](#_ENREF_14), [15](#_ENREF_15), [22-25](#_ENREF_22)] |
|  | Importance of stranger danger | - | Negative | [[3](#_ENREF_3), [7](#_ENREF_7), [9](#_ENREF_9), [14](#_ENREF_14), [15](#_ENREF_15), [18](#_ENREF_18), [24](#_ENREF_24), [26](#_ENREF_26)] |
|  | Importance of traffic safety | - | Negative | [[1](#_ENREF_1), [3](#_ENREF_3), [12](#_ENREF_12), [14](#_ENREF_14), [15](#_ENREF_15), [18](#_ENREF_18), [24](#_ENREF_24), [26](#_ENREF_26), [27](#_ENREF_27)] |
|  | Importance of convenience | - | Negative | [[6](#_ENREF_6), [9](#_ENREF_9), [16](#_ENREF_16), [18](#_ENREF_18), [19](#_ENREF_19), [25](#_ENREF_25), [27](#_ENREF_27), [28](#_ENREF_28)] |
|  | Importance of distance to school | - | Negative | [[1](#_ENREF_1), [6](#_ENREF_6), [7](#_ENREF_7), [9](#_ENREF_9), [14-16](#_ENREF_14), [18](#_ENREF_18), [26-29](#_ENREF_26)] |
| Child Characteristics | Year | - | Positive | [[1](#_ENREF_1), [6](#_ENREF_6), [15](#_ENREF_15), [30](#_ENREF_30)] |
|  | Sex (reference category: male) | - | Negative | [[1](#_ENREF_1), [2](#_ENREF_2), [31](#_ENREF_31)] |
|  | Ethnicity (reference category: New Zealand European) | - | Negative | [[2](#_ENREF_2)] |
|  | Physical activity | - | Positive | [[32-36](#_ENREF_32)] |
| Child Beliefs | Traffic safety | - | Positive | [[1](#_ENREF_1), [12](#_ENREF_12), [14](#_ENREF_14), [18](#_ENREF_18), [24](#_ENREF_24), [26](#_ENREF_26)] |
|  | Neighbourhood safety | - | Positive | [[14](#_ENREF_14), [15](#_ENREF_15), [18](#_ENREF_18), [24](#_ENREF_24), [26](#_ENREF_26), [37](#_ENREF_37), [38](#_ENREF_38)] |
|  | Independent mobility | - | Positive | [[9](#_ENREF_9), [19](#_ENREF_19), [20](#_ENREF_20)] |
| *Observed variables deleted after model modification | | | | |

**References**

1. Rothman L, Macpherson AK, Ross T, Buliung RN. The decline in active school transportation (AST): a systematic review of the factors related to AST and changes in school transport over time in North America. Prev Med. 2018; doi:10.1016/j.ypmed.2017.11.018.

2. Ikeda E, Stewart T, Garrett N, Egli V, Mandic S, Hosking J, Witten K, Hawley G, Tautolo ES, Rodda J *et al*. Built environment associates of active school travel in New Zealand children and youth: a systematic meta-analysis using individual participant data. J Transp Health. 2018; doi:10.1016/j.jth.2018.04.007.

3. Stewart O. Findings from research on active transportation to school and implications for Safe Routes to School programs. J Plan Lit. 2011; doi:10.1177/0885412210385911.

4. Stewart O, Vernez Moudon A, Claybrooke C. Common ground: eight factors that influence walking and biking to school. Transp Policy. 2012; doi:10.1016/j.tranpol.2012.06.016.

5. Giles-Corti B, Wood G, Pikora T, Learnihan V, Bulsara M, Van Niel K, Timperio A, McCormack G, Villanueva K. School site and the potential to walk to school: the impact of street connectivity and traffic exposure in school neighborhoods. Health Place. 2011; doi:10.1016/j.healthplace.2010.12.011.

6. Lee C, Zhu X, Yoon J, Varni JW. Beyond distance: Children’s school travel mode choice. Ann Behav Med. 2013; doi:10.1007/s12160-012-9432-z.

7. Lee C, Yoon J, Zhu X. From sedentary to active school commute: multi-level factors associated with travel mode shifts. Prev Med. 2017; doi:10.1016/j.ypmed.2016.10.018.

8. Wong BY-M, Faulkner GEJ, Buliung RN. GIS measured environmental correlates of active school transport: a systematic review of 14 studies. Int J Behav Nutr Phys Act. 2011; doi:10.1186/1479-5868-8-39.

9. Mitra R. Independent mobility and mode choice for school transportation: a review and framework for future research. Transp Rev. 2013; doi:10.1080/01441647.2012.743490.

10. Oliver M, Badland HM, Mavoa S, Witten K, Kearns R, Ellaway A, Hinckson E, Mackay L, Schluter PJ. Environmental and socio-demographic associates of children’s active transport to school: a cross-sectional investigation from the URBAN Study. Int J Behav Nutr Phys Act. 2014; doi:10.1186/1479-5868-11-70.

11. Mehdizadeh M, Fallah Zavareh M, Nordfjaern T. School travel mode use: direct and indirect effects through parental attitudes and transport priorities. Transportmetrica A: Transport Science. 2018; doi:10.1080/23249935.2018.1529838.

12. Aarts M-J, Mathijssen JJP, van Oers JAM, Schuit AJ. Associations between environmental characteristics and active commuting to school among children: a cross-sectional study. Int J Behav Med. 2013;20:538-555.

13. Ross A, Rodriguez A, Searle M. Associations between the physical, sociocultural, and safety environments and active transportation to school. Am J Health Ed. 2017; doi:10.1080/19325037.2017.1292877.

14. Ikeda E, Hinckson E, Witten K, Smith M. Associations of children’s active school travel with perceptions of the physical environment and characteristics of the social environment: A systematic review. Health Place. In second review; doi

15. Yu C-Y, Zhu X. From attitude to action: what shapes attitude toward walking to/from school and how does it influence actual behaviors? Prev Med. 2016; doi:10.1016/j.ypmed.2016.06.036.

16. Yu C-Y, Zhu X. Impacts of residential self-selection and built environments on children’s walking-to-school behaviors. Environ Behav. 2015; doi:10.1177/0013916513500959.

17. Mehdizadeh M, Nordfjaern T, Mamdoohi A. The role of socio-economic, built environment and psychological factors in parental mode choice for their children in an Iranian setting. Transportation. 2018; doi:10.1007/s11116-016-9737-z.

18. Ahlport KN, Linnan L, Vaughn A, Evenson KR, Ward DS. Barriers to and facilitators of walking and bicycling to school: formative results from the non-motorized travel study. Health Educ Behav. 2008; doi:10.1177/1090198106288794.

19. Westman J, Friman M, Olsson LE. What drives them to drive? Parents' reasons for choosing the car to take their children to school. Front Psychol. 2017; doi:10.3389/fpsyg.2017.01970.

20. Buliung RN, Larsen K, Faulkner G, Ross T. Children’s independent mobility in the City of Toronto, Canada. Travel Behav Soc. 2017; doi:10.1016/j.tbs.2017.06.001.

21. McDonald NC. Household interactions and children’s school travel: the effect of parental work patterns on walking and biking to school. J Transp Geogr. 2008;16:324-331.

22. Waygood EOD, Friman M, Olsson LE, Taniguchi A. Transport and child well-being: an integrative review. Travel Behav Soc. 2017;9:32-49.

23. Panter JR, Jones AP, van Sluijs EMF. Environmental determinants of active travel in youth: a review and framework for future research. Int J Behav Nutr Phys Act. 2008; doi:10.1186/1479-5868-5-34.

24. Timperio A, Ball K, Salmon J, Roberts R, Giles-Corti B, Simmons D, Baur LA, Crawford D. Personal, family, social, and environmental correlates of active commuting to school. Am J Prev Med. 2006;30:45-51.

25. Eyler A, Baldwin J, Carnoske C, Nickelson J, Troped P, Steinman L, Pluto D, Litt J, Evenson K, Terpstra J *et al*. Parental involvement in active transport to school initiatives. Am J Health Ed. 2008; doi:10.1080/19325037.2008.10599029.

26. Chillón P, Hales D, Vaughn A, Gizlice Z, Ni A, Ward DS. A cross-sectional study of demographic, environmental and parental barriers to active school travel among children in the United States. Int J Behav Nutr Phys Act. 2014; doi:10.1186/1479-5868-11-61.

27. Ahern SM, Arnott B, Chatterton T, de Nazelle A, Kellar I, McEachan RRC. Understanding parents' school travel choices: a qualitative study using the Theoretical Domains Framework. J Transp Health. 2017; doi:10.1016/j.jth.2016.11.001.

28. Panter JR, Corder K, Griffin SJ, Jones AP, van Sluijs EMF. Individual, socio-cultural and environmental predictors of uptake and maintenance of active commuting in children: longitudinal results from the SPEEDY study. Int J Behav Nutr Phys Act. 2013;10:1-12.

29. Zhu X, Lee C, Lu Z, Yu C-Y, College of Architecture, Texas A&M University: Walkable distance for elementary school children and impacts of individualized objective environment on active school transportation. In: *9th Active Living Research Annual Conference: March 12-14 2012; San Diego, CA*; 2012.

30. McDonald NC, Brown AL, Marchetti LM, Pedroso MS. U.S. school travel, 2009: An assessment of trends. Am J Prev Med. 2011;41:146-151.

31. McMillan T, Day K, Boarnet M, Alfonzo M, Anderson C. Johnny walks to school - Does Jane? Sex differences in children's active travel to school. Children, Youth and Environments. 2006;16:75-89.

32. Faulkner GEJ, Buliung RN, Flora PK, Fusco C. Active school transport, physical activity levels and body weight of children and youth: a systematic review. Prev Med. 2009;48:3-8.

33. Martin A, Boyle J, Corlett F, Kelly P, Reilly JJ. Contribution of walking to school to individual and population moderate-vigorous intensity physical activity: systematic review and meta-analysis. Pediatr Exerc Sci. 2016;28:353-363.

34. Lee MC, Orenstein MR, Richardson MJ. Systematic review of active commuting to school and children's physical activity and weight. J Phys Act Health. 2008;5:930-949.

35. Stewart T, Duncan S, Schipperijn J. Adolescents who engage in active school transport are also more active in other contexts: a space-time investigation. Health Place. 2017;43:25-32.

36. Oliver M, Mavoa S, Badland HM, Parker K, Donovan P, Kearns RA, Lin E-Y, Witten K. Associations between the neighbourhood built environment and out of school physical activity and active travel: an examination from the Kids in the City study. Health Place. 2015; doi:10.1016/j.healthplace.2015.09.005.

37. Pont K, Ziviani J, Wadley D, Bennett S, Abbott R. Environmental correlates of children's active transportation: a systematic literature review. Health Place. 2009; doi:10.1016/j.healthplace.2009.02.002.

38. Sirard JR, Slater ME. Walking and bicycling to school: a review. Am J Lifestyle Med. 2008; doi:10.1177/1559827608320127.
