# Supplementary material for: Assessment of direct and indirect associations between children active school travel and environmental, household and child factors using structural equation modelling
Source: Int J Behav Nutr Phys Act. 2019 Apr 5;16:32. doi: 10.1186/s12966-019-0794-5 (PMC6451289; doi:10.1186/s12966-019-0794-5)
Supplement: Supplementary file 2 — Hypothesised indirect relationships between children’s school travel behaviour and the built environment, the social environment, household and child characteristics, and household and child beliefs. (DOCX 95.1 kb) [file 12966_2019_794_MOESM2_ESM.docx]

# Additional file 2

Hypothesised indirect relationships between children’s school travel behaviour and the built environment, the social environment, household and child characteristics, and household and child beliefs

| **Independent variable** | |  | **Dependent variable (Mediator)** | | **References** |
| --- | --- | --- | --- | --- | --- |
| Variable† | Domain |  | Variable† | Domain |  |
| *Active mobility environment* | BE |  | Distance to school | BE | [[1](#_ENREF_1), [2](#_ENREF_2)] |
| *Active mobility environment* | BE |  | *Neighbourhood social environment* | SE | [[3-6](#_ENREF_3)] |
| *Active mobility environment* | BE |  | Importance of distance to school | HB | [[7](#_ENREF_7), [8](#_ENREF_8)] |
| *Active mobility environment* | BE |  | Importance of traffic safety | HB | [[8](#_ENREF_8), [9](#_ENREF_9)] |
| *Active mobility environment* | BE |  | Importance of stranger danger | HB | [[5](#_ENREF_5), [8](#_ENREF_8)] |
| *Active mobility environment* | BE |  | Traffic safety | CB | [[7](#_ENREF_7), [8](#_ENREF_8)] |
| *Active mobility environment* | BE |  | Independent mobility | CB | [[10](#_ENREF_10), [11](#_ENREF_11)] |
| Distance to school | BE |  | Independent mobility | CB | [[11-13](#_ENREF_11)] |
| *Neighbourhood social environment* | SE |  | Importance of stranger danger | HB | [[5](#_ENREF_5), [14](#_ENREF_14)] |
| *Neighbourhood social environment* | SE |  | Importance of social interaction | HB | [[14-16](#_ENREF_14)] |
| *Neighbourhood social environment* | SE |  | Independent mobility | CB | [[11](#_ENREF_11), [12](#_ENREF_12), [14](#_ENREF_14), [17-19](#_ENREF_17)] |
| *Neighbourhood social environment* | SE |  | Neighbourhood safety | CB | [[12](#_ENREF_12), [14](#_ENREF_14)] |
| Number of children | HC |  | Car ownership | HC | [[20](#_ENREF_20), [21](#_ENREF_21)] |
| Number of children* | HC |  | Parent employment* | HC | [[22](#_ENREF_22)] |
| Number of adults* | HC |  | Car ownership* | HC | [[20](#_ENREF_20), [21](#_ENREF_21)] |
| Parent education* | HC |  | Car ownership* | HC | [[21](#_ENREF_21)] |
| Parent education* | HC |  | Parent employment* | HC | [[23](#_ENREF_23)] |
| Parent employment* | HC |  | Car ownership* | HC | [[21](#_ENREF_21)] |
| Number of children | HC |  | Importance of convenience | HB | [[24-26](#_ENREF_24)] |
| Importance of stranger danger | HB |  | Importance of traffic safety | HB | [[2](#_ENREF_2), [5](#_ENREF_5), [27](#_ENREF_27), [28](#_ENREF_28)] |
| Importance of stranger danger | HB |  | Independent mobility | CB | [[2](#_ENREF_2), [5](#_ENREF_5), [11](#_ENREF_11), [13](#_ENREF_13), [18](#_ENREF_18), [19](#_ENREF_19), [29](#_ENREF_29)] |
| Importance of traffic safety | HB |  | Independent mobility | CB | [[2](#_ENREF_2), [5](#_ENREF_5), [11](#_ENREF_11), [13](#_ENREF_13), [14](#_ENREF_14), [18](#_ENREF_18), [29](#_ENREF_29)] |
| Independent mobility | CB |  | Neighbourhood safety | CB | [[11](#_ENREF_11), [18](#_ENREF_18), [29](#_ENREF_29)] |
| Year | CC |  | Distance to school | BE | [[13](#_ENREF_13), [30](#_ENREF_30)] |
| Year | CC |  | Importance of social interaction | HB | [[31](#_ENREF_31)] |
| Year | CC |  | Importance of stranger danger | HB | [[2](#_ENREF_2), [5](#_ENREF_5), [13](#_ENREF_13), [14](#_ENREF_14)] |
| Year | CC |  | Importance of traffic safety | HB | [[2](#_ENREF_2), [13](#_ENREF_13), [14](#_ENREF_14)] |
| Year | CC |  | Importance of convenience | HB | [[32-35](#_ENREF_32)] |
| Year | CC |  | Importance of distance to school | HB | [[13](#_ENREF_13), [30](#_ENREF_30)] |
| Year | CC |  | Traffic safety | CB | [[36](#_ENREF_36)] |
| Year | CC |  | Neighbourhood safety | CB | [[36](#_ENREF_36)] |
| Year | CC |  | Independent mobility | CB | [[11](#_ENREF_11), [12](#_ENREF_12), [14](#_ENREF_14), [24](#_ENREF_24), [37](#_ENREF_37)] |
| Year | CC |  | Physical activity | CC | [[38](#_ENREF_38), [39](#_ENREF_39)] |
| Sex | CC |  | Importance of social interaction | HB | [[31](#_ENREF_31)] |
| Sex | CC |  | Importance of stranger danger | HB | [[2](#_ENREF_2), [13](#_ENREF_13), [14](#_ENREF_14)] |
| Sex | CC |  | Importance of traffic safety | HB | [[2](#_ENREF_2), [13](#_ENREF_13), [14](#_ENREF_14)] |
| Sex | CC |  | Importance of distance to school | HB | [[30](#_ENREF_30)] |
| Sex | CC |  | Importance of convenience | HB | [[32-35](#_ENREF_32)] |
| Sex | CC |  | Traffic safety | CB | [[36](#_ENREF_36)] |
| Sex | CC |  | Neighbourhood safety | CB | [[36](#_ENREF_36)] |
| Sex | CC |  | Independent mobility | CB | [[11](#_ENREF_11), [12](#_ENREF_12), [37](#_ENREF_37)] |
| Sex | CC |  | Physical activity | CC | [[38](#_ENREF_38), [39](#_ENREF_39)] |
| Ethnicity | CC |  | Physical activity | CC | [[40](#_ENREF_40), [41](#_ENREF_41)] |
| Physical activity | CC |  | Importance of social interaction | HB | [[6](#_ENREF_6), [42](#_ENREF_42)] |
| BE = built environment. CB = child beliefs. CC = child characteristics. HB = household beliefs. HC = household characteristics. SE = social environment. †Observed variables; *Latent variables*. *Dependent relationships deleted after model modification | | | | | |

**References**

1. Wong BY-M, Faulkner GEJ, Buliung RN. GIS measured environmental correlates of active school transport: A systematic review of 14 studies. Int J Behav Nutr Phys Act. 2011; doi:10.1186/1479-5868-8-39.

2. Stewart O, Vernez Moudon A, Claybrooke C. Common ground: Eight factors that influence walking and biking to school. Transp Policy. 2012; doi:10.1016/j.tranpol.2012.06.016.

3. Jun H-J, Hur M. The relationship between walkability and neighborhood social environment: The importance of physical and perceived walkability. Appl Geogr. 2015; doi:https://doi.org/10.1016/j.apgeog.2015.04.014.

4. Leyden KM. Social capital and the built environment: The importance of walkable neighborhoods. Am J Public Health. 2003; doi:10.2105/ajph.93.9.1546.

5. Johansson M. Environment and parental factors as determinants of mode for children's leisure travel. J Environ Psychol. 2006; doi:10.1016/j.jenvp.2006.05.005.

6. Zhu X, Yu C-Y, Lee C, Lu Z, Mann G. A retrospective study on changes in residents' physical activities, social interactions, and neighborhood cohesion after moving to a walkable community. Prev Med. 2014; doi:10.1016/j.ypmed.2014.08.013.

7. Giles-Corti B, Wood G, Pikora T, Learnihan V, Bulsara M, Van Niel K, Timperio A, McCormack G, Villanueva K. School site and the potential to walk to school: The impact of street connectivity and traffic exposure in school neighborhoods. Health Place. 2011; doi:10.1016/j.healthplace.2010.12.011.

8. Gallimore JM, Brown BB, Werner CM. Walking routes to school in new urban and suburban neighborhoods: An environmental walkability analysis of blocks and routes. J Environ Psychol. 2011; doi:10.1016/j.jenvp.2011.01.001.

9. Rojas Lopez MC, Wong YD. Children’s active trips to school: A review and analysis. International Journal of Urban Sustainable Development. 2017; doi:10.1080/19463138.2016.1264405.

10. Villanueva K, Giles-Corti B, Bulsara M, Trapp G, Timperio A, McCormack G, Van Niel K. Does the walkability of neighbourhoods affect children's independent mobility, independent of parental, socio-cultural and individual factors? Children's Geogr. 2014; doi:10.1080/14733285.2013.812311.

11. Marzi I, Demetriou Y, Reimers AK. Social and physical environmental correlates of independent mobility in children: a systematic review taking sex/gender differences into account. Int J Health Geogr. 2018; doi:10.1186/s12942-018-0145-9.

12. Lin E-Y, Witten K, Oliver M, Carroll P, Asiasiga L, Badland H, Parker K. Social and built-environment factors related to children's independent mobility: The importance of neighbourhood cohesion and connectedness. Health Place. 2017; doi:10.1016/j.healthplace.2017.05.002.

13. Westman J, Friman M, Olsson LE. What drives them to drive? Parents' reasons for choosing the car to take their children to school. Front Psychol. 2017; doi:10.3389/fpsyg.2017.01970.

14. Waygood EOD, Friman M, Olsson LE, Taniguchi A. Transport and child well-being: An integrative review. Travel Behav Soc. 2017; doi:10.1016/j.tbs.2017.04.005.

15. Witten K, Kearns R, Carroll P. Urban inclusion as wellbeing: Exploring children's accounts of confronting diversity on inner city streets. Soc Sci Med. 2015; doi:10.1016/j.socscimed.2015.01.016.

16. Pacilli MG, Giovannelli I, Prezza M, Augimeri ML. Children and the public realm: antecedents and consequences of independent mobility in a group of 11–13-year-old Italian children. Children's Geogr. 2013; doi:10.1080/14733285.2013.812277.

17. Weller S, Bruegel I. Children's `place' in the development of neighbourhood social capital. Urban Studies. 2009; doi:10.1177/0042098008100998.

18. Wolfe MK, McDonald NC. Association between neighborhood social environment and children's independent mobility. J Phys Act Health. 2016; doi:10.1123/jpah.2015-0662.

19. Foster S, Villanueva K, Wood L, Christian H, Giles-Corti B. The impact of parents’ fear of strangers and perceptions of informal social control on children's independent mobility. Health Place. 2014; doi:10.1016/j.healthplace.2013.11.006.

20. Dargay JM. The effect of income on car ownership: evidence of asymmetry. Transp Res Part A Policy Pract. 2001; doi:10.1016/S0965-8564(00)00018-5.

21. 2013 Census quick stats about transport and communications: Number of motor vehicles. <http://archive.stats.govt.nz/Census/2013-census/profile-and-summary-reports/quickstats-transport-comms/number-motor-vehicles.aspx>. 2018.

22. Stats New Zealand. New Zealand's children. 1999. <http://archive.stats.govt.nz/browse_for_stats/people_and_communities/Children/nzs-children.aspx>.

23. Ministry of Education. Looking at the employment outcomes of tertiary education: New data on the earnings of young graduates. 2013. https://[www.educationcounts.govt.nz/__data/assets/pdf_file/0020/143561/Looking-at-the-employment-outcomes-of-tertiary-education-ii.pdf](http://www.educationcounts.govt.nz/__data/assets/pdf_file/0020/143561/Looking-at-the-employment-outcomes-of-tertiary-education-ii.pdf).

24. Mitra R. Independent mobility and mode choice for school transportation: A review and framework for future research. Transp Rev. 2013; doi:10.1080/01441647.2012.743490.

25. Ahern SM, Arnott B, Chatterton T, de Nazelle A, Kellar I, McEachan RRC. Understanding parents' school travel choices: A qualitative study using the Theoretical Domains Framework. J Transp Health. 2017; doi:10.1016/j.jth.2016.11.001.

26. Ahlport KN, Linnan L, Vaughn A, Evenson KR, Ward DS. Barriers to and facilitators of walking and bicycling to school: Formative results from the non-motorized travel study. Health Educ Behav. 2008; doi:10.1177/1090198106288794.

27. McMillan TE. The relative influence of urban form on a child’s travel mode to school. Transp Res Part A Policy Pract. 2007; doi:10.1016/j.tra.2006.05.011.

28. Timperio A, Crawford D, Telford A, Salmon J. Perceptions about the local neighborhood and walking and cycling among children. Prev Med. 2004; doi:10.1016/j.ypmed.2003.09.026.

29. Shaw B, Bicket M, Elliot B, Fagan-Watson B, Mocca E, Hillman M. Children's independent mobility: An international comparison and recommendations for action. 2015. <http://www.psi.org.uk/children_mobility>.

30. Duncan S, White K, Mavoa S, Stewart T, Hinckson E, Schofield G. Active transport, physical activity, and distance between home and school in children and adolescents. J Phys Act Health. 2016; doi:10.1123/jpah.2015-0054.

31. Aarts M-J, Mathijssen JJP, van Oers JAM, Schuit AJ. Associations between environmental characteristics and active commuting to school among children: A cross-sectional study. Int J Behav Med. 2013; doi:10.1007/s12529-012-9271-0.

32. McDonald NC. Household interactions and children’s school travel: the effect of parental work patterns on walking and biking to school. J Transp Geogr. 2008; doi:10.1016/j.jtrangeo.2008.01.002.

33. Schlossberg M, Greene J, Phillips PP, Johnson B, Parker B. School trips: Effects of urban form and distance on travel mode. J Am Plan Assoc. 2006; doi:10.1080/01944360608976755.

34. Buliung R, Faulkner G, Beesley T, Kennedy J. School travel planning: Mobilizing school and community resources to encourage active school transportation. J Sch Health. 2011; doi:10.1111/j.1746-1561.2011.00647.x.

35. Faulkner GEJ, Richichi V, Buliung RN, Fusco C, Moola F. What’s “quickest and easiest?”: Parental decision making about school trip mode. Int J Behav Nutr Phys Act. 2010; doi:10.1186/1479-5868-7-62.

36. Mullan E. Do you think that your local area is a good place for young people to grow up? The effects of traffic and car parking on young people's views. Health Place. 2003; doi:10.1016/S1353-8292(02)00069-2.

37. Buliung RN, Larsen K, Faulkner G, Ross T. Children’s independent mobility in the City of Toronto, Canada. Travel Behav Soc. 2017; doi:10.1016/j.tbs.2017.06.001.

38. Martin A, Boyle J, Corlett F, Kelly P, Reilly JJ. Contribution of walking to school to individual and population moderate-vigorous intensity physical activity: Systematic review and meta-analysis. Pediatr Exerc Sci. 2016; doi:10.1123/pes.2015-0207.

39. Lee MC, Orenstein MR, Richardson MJ. Systematic review of active commuting to school and children's physical activity and weight. J Phys Act Health. 2008;5:930-949.

40. Oliver M, Mavoa S, Badland HM, Parker K, Donovan P, Kearns RA, Lin E-Y, Witten K. Associations between the neighbourhood built environment and out of school physical activity and active travel: An examination from the Kids in the City study. Health Place. 2015; doi:10.1016/j.healthplace.2015.09.005.

41. Oliver M, Parker K, Witten K, Mavoa S, Badland HM, Donovan P, Chaudhury M, Kearns RA. Children's out-of-school independently mobile tips, active travel, and physical activity: A cross-sectional examination from the Kids in the City Study. J Phys Act Health. 2016; doi:10.1123/jpah.2015-0043.

42. Carver A, Timperio A, Crawford D. Playing it safe: The influence of neighbourhood safety on children's physical activity—A review. Health Place. 2008; doi:10.1016/j.healthplace.2007.06.004.
