# Supplementary material for: Assessment of direct and indirect associations between children active school travel and environmental, household and child factors using structural equation modelling
Source: Int J Behav Nutr Phys Act. 2019 Apr 5;16:32. doi: 10.1186/s12966-019-0794-5 (PMC6451289; doi:10.1186/s12966-019-0794-5)
Supplement: Supplementary file 3 — Table S1. Information about observed variables and their descriptive statistics. (N = 1085) (DOCX 63.7 kb) [file 12966_2019_794_MOESM3_ESM.docx]

# Table 1

Information about observed variables and their descriptive statistics (N = 1085)

| **Observed variable** | **Latent variable** | **Description** | **Data source** | **Variable type** | **Missing (%)** | **Measurement scale** | **Descriptive statistics†** |
| --- | --- | --- | --- | --- | --- | --- | --- |
| *School Travel Mode* | | | | | | | |
| Active school travel | - | How do you usually get to school? | SoftGIS | Binary | 0.0 | 0 = passive travel (i.e., car, public transport) | 58.0% |
|  |  |  |  |  |  | Car | 46.1% |
|  |  |  |  |  |  | Public transport | 11.9% |
|  |  |  |  |  | 0.0 | 1 = active travel (i.e., walk, bike, scooter, skateboard) | 42.0% |
|  |  |  |  |  |  | Walk | 34.4% |
|  |  |  |  |  |  | Bike | 3.9% |
|  |  |  |  |  |  | Scooter, skateboard | 3.8% |
| *Child Characteristics* | | | | | | | |
| Year | - | Child's school year | School/Parent consent form | Continuous | 0.0 | 5 = Year 5 | 24.5% |
|  |  |  |  |  |  | 6 = Year 6 | 26.4% |
|  |  |  |  |  |  | 7 = Year 7 | 24.2% |
|  |  |  |  |  |  | 8 = Year 8 | 24.9% |
| Sex | - | Child's sex | School/Parent consent form | Binary | 0.0 | 0 = male | 49.0% |
|  |  |  |  |  |  | 1 = female | 51.0% |
| Ethnicity | - | New Zealand (NZ) European | School/CATI | Binary* | 1.2 | 0 = Māori/Pacific/Asian/other | 46.1% |
|  |  |  |  |  |  | 1 = NZ European | 52.7% |
|  |  | Māori | School/CATI | Binary* | 1.2 | 0 = NZ European/Pacific/Asian/other | 85.9% |
|  |  |  |  |  |  | 1 = Māori | 12.9% |
|  |  | Pacific | School/CATI | Binary* | 1.2 | 0 = NZ European/Māori/Asian/other | 84.5% |
|  |  |  |  |  |  | 1 = Pacific | 15.3% |
|  |  | Asian | School/CATI | Binary* | 1.2 | 0 = NZ European/Māori/Pacific/other | 83.8% |
|  |  |  |  |  |  | 1 = Asian | 15.0% |
| Physical activity | - | Percentage of time spent in overall (light + moderate + vigorous) physical activity during the morning (8:00-9:00 am) commute | Accelerometer | Continuous | 8.4 | - | 8.8 ± 3.0 |
| *Child Beliefs* | | | | | | | |
| Traffic safety | - | Summed scores of 2 items: | SoftGIS | Continuous | 0.4 | - | 4.8 ± 1.4 |
|  |  | 1. The roads around my school are busy with traffic before and after school. | SoftGIS | Ordinal | 0.2 | 1 = all of the time | 13.0% |
|  |  |  |  |  |  | 2 = most of the time | 40.7% |
|  |  |  |  |  |  | 3 = sometimes | 37.2% |
|  |  |  |  |  |  | 4 = hardly ever/never | 8.8% |
|  |  | 2. The roads around my school are full of parked cars before and after school. | SoftGIS | Ordinal | 0.4 | 1 = all of the time | 17.1% |
|  |  |  |  |  |  | 2 = most of the time | 36.2% |
|  |  |  |  |  |  | 3 = sometimes | 35.9% |
|  |  |  |  |  |  | 4 = hardly ever/never | 10.3% |
| Neighbourhood safety | - | Summed scores of 2 items: | SoftGIS | Continuous | 0.4 | - | 6.2 ± 1.3 |
|  |  | 1. If I am out with an adult, I feel safe in my neighbourhood. | SoftGIS | Ordinal | 0.4 | 1 = hardly ever/never/do not go out with an adult in the neighbourhood | 2.0% |
|  |  |  |  |  |  | 2 = sometimes | 8.0% |
|  |  |  |  |  |  | 3 = most of the time | 21.2% |
|  |  |  |  |  |  | 4 = all of the time | 68.4% |
|  |  | 2. If I go out without an adult, I feel safe in my neighbourhood. | SoftGIS | Ordinal | 0.3 | 1 = hardly ever/never/do not go out without an adult in the neighbourhood | 17.9% |
|  |  |  |  |  |  | 2 = sometimes | 27.3% |
|  |  |  |  |  |  | 3 = most of the time | 33.2% |
|  |  |  |  |  |  | 4 = all of the time | 21.4% |
| Independent mobility | - | Summed scores of 3 items: | SoftGIS | Continuous | 1.0 | - | 4.6 ± 1.0 |
|  |  | 1. Are you allowed to cross main roads on your own? | SoftGIS | Binary | 0.4 | 1 = no | 32.3% |
|  |  |  |  |  |  | 2 = yes | 67.4% |
|  |  | 2. Are you allowed to go on local buses or trains or ferries on your own? | SoftGIS | Binary | 0.8 | 1 = no | 71.2% |
|  |  |  |  |  |  | 2 = yes | 27.9% |
|  |  | 3. If you have a bicycle, are you allowed to ride it to go to places? | SoftGIS | Binary | 0.6 | 1 = no/do not have a bicycle | 40.5% |
|  |  |  |  |  |  | 2 = yes | 58.9% |
| *Household Characteristics* | | | | | | | |
| Education | - | What is your highest academic qualification? | CATI | Binary | 18.8 | 0 = Certificate (levels 1-6), Diploma or lower | 51.2% |
|  |  |  |  |  |  | 1 = Bachelor's degree or higher | 30.0% |
| Employment | - | Which one best describes your main current employment situation? Full-time paid work. | CATI | Binary* | 18.2 | 0 = part-time/other | 41.8% |
|  |  |  |  |  |  | 1 = full-time | 40.0% |
|  |  | Which one best describes your main current employment situation? Part-time paid work. | CATI | Binary* | 18.2 | 0 = full-time/other | 56.8% |
|  |  |  |  |  |  | 1 = part-time | 25.0% |
| Number of adults | - | How many adults, including yourself, live in your household? | CATI | Ordinal | 18.2 | 1 = 1 adult | 9.1% |
|  |  |  |  |  |  | 2 = 2 adults | 56.5% |
|  |  |  |  |  |  | 3 = 3 adults | 9.2% |
|  |  |  |  |  |  | 4 = 4 adults | 4.7% |
|  |  |  |  |  |  | 5 = greater than or equal to 5 adults | 2.3% |
| Number of children | - | How many other children under 18 live in your household? | CATI | Ordinal | 18.2 | 0 = no other children | 12.1% |
|  |  |  |  |  |  | 1 = 1 child | 36.2% |
|  |  |  |  |  |  | 2 = 2 children | 21.8% |
|  |  |  |  |  |  | 3 = 3 children | 7.6% |
|  |  |  |  |  |  | 4 = greater than or equal to 4 children | 4.1% |
| Car ownership | - | How many working cars are available to your household? | CATI | Binary | 18.2 | 0 = less than or equal to 1 car | 18.1% |
|  |  |  |  |  |  | 1 = greater than or equal to 2 cars | 63.8% |
| *Household Beliefs* | | | | | | | |
| Distance to school | - | What are the main reasons your child gets to school by (travel mode to school)? How important would you say this reason (i.e., distance to school) when deciding how your child gets to school? | CATI | Ordinal | 21.7 | 1 = not main reason | 35.0% |
|  |  |  |  |  |  | 2 = main reason, but not important | 0.6% |
|  |  |  |  |  |  | 3 = main reason, and a little bit important | 0.8% |
|  |  |  |  |  |  | 4 = main reason, and important | 6.3% |
|  |  |  |  |  |  | 5 = main reason, and very important | 35.6% |
| Traffic safety | - | What are the main reasons your child gets to school by (travel mode to school)? How important would you say this reason (i.e., traffic safety) when deciding how your child gets to school? | CATI | Ordinal | 14.9 | 1 = not main reason | 78.5% |
|  |  |  |  |  |  | 2 = main reason, but not important | 0.1% |
|  |  |  |  |  |  | 3 = main reason, and a little bit important | 0.1% |
|  |  |  |  |  |  | 4 = main reason, and important | 0.6% |
|  |  |  |  |  |  | 5 = main reason, and very important | 5.7% |
| Stranger danger | - | What are the main reasons your child gets to school by (travel mode to school)? How important would you say this reason (i.e., stranger danger) when deciding how your child gets to school? | CATI | Ordinal | 14.6 | 1 = not main reason | 79.6% |
|  |  |  |  |  |  | 2 = main reason, but not important | 0.0% |
|  |  |  |  |  |  | 3 = main reason, and a little bit important | 0.2% |
|  |  |  |  |  |  | 4 = main reason, and important | 0.6% |
|  |  |  |  |  |  | 5 = main reason, and very important | 5.0% |
| Convenience | - | What are the main reasons your child gets to school by (travel mode to school)? How important would you say this reason (i.e., convenience) when deciding how your child gets to school? | CATI | Ordinal | 17.2 | 1 = not main reason | 56.0% |
|  |  |  |  |  |  | 2 = main reason, but not important | 0.6% |
|  |  |  |  |  |  | 3 = main reason, and a little bit important | 2.8% |
|  |  |  |  |  |  | 4 = main reason, and important | 7.9% |
|  |  |  |  |  |  | 5 = main reason, and very important | 15.4% |
| Social interaction | - | What are the main reasons your child gets to school by (travel mode to school)? How important would you say this reason (i.e., social interaction) when deciding how your child gets to school? | CATI | Ordinal | 14.3 | 1 = not main reason | 80.8% |
|  |  |  |  |  |  | 2 = main reason, but not important | 0.0% |
|  |  |  |  |  |  | 3 = main reason, and a little bit important | 0.7% |
|  |  |  |  |  |  | 4 = main reason, and important | 1.8% |
|  |  |  |  |  |  | 5 = main reason, and very important | 2.4% |
| *Social environment* | | | | | | | |
| Neighbourhood safety | Neighbourhood social environment | Summed scores of 9 items: | CATI | Continuous | 27.6 | - | 33.4 ± 4.8 |
|  |  | 1. There are safe places for children to play in our neighbourhood. | CATI | Ordinal | 19.2 | 1 = strongly disagree | 1.0% |
|  |  |  |  |  |  | 2 = disagree | 11.7% |
|  |  |  |  |  |  | 3 = neither agree nor disagree | 5.9% |
|  |  |  |  |  |  | 4 = agree | 47.7% |
|  |  |  |  |  |  | 5 = strongly agree | 14.5% |
|  |  | 2. It’s a good place to bring up children. | CATI | Ordinal | 18.8 | 1 = strongly disagree | 0.6% |
|  |  |  |  |  |  | 2 = disagree | 2.7% |
|  |  |  |  |  |  | 3 = neither agree nor disagree | 5.1% |
|  |  |  |  |  |  | 4 = agree | 48.3% |
|  |  |  |  |  |  | 5 = strongly agree | 24.6% |
|  |  | 3. I feel safe walking down my street after dark. | CATI | Ordinal | 19.5 | 1 = strongly disagree | 3.6% |
|  |  |  |  |  |  | 2 = disagree | 18.4% |
|  |  |  |  |  |  | 3 = neither agree nor disagree | 7.0% |
|  |  |  |  |  |  | 4 = agree | 41.6% |
|  |  |  |  |  |  | 5 = strongly agree | 9.9% |
|  |  | 4. I worry about the number of crimes committed in our neighbourhood. | CATI | Ordinal | 19.3 | 1 = strongly agree | 6.0% |
|  |  |  |  |  |  | 2 = agree | 25.4% |
|  |  |  |  |  |  | 3 = neither agree nor disagree | 11.2% |
|  |  |  |  |  |  | 4 = disagree | 34.1% |
|  |  |  |  |  |  | 5 = strongly disagree | 4.1% |
|  |  | 5. Graffiti and vandalism are problems. | CATI | Ordinal | 18.7 | 1 = strongly agree | 2.9% |
|  |  |  |  |  |  | 2 = agree | 14.4% |
|  |  |  |  |  |  | 3 = neither agree nor disagree | 5.3% |
|  |  |  |  |  |  | 4 = disagree | 52.7% |
|  |  |  |  |  |  | 5 = strongly disagree | 6.1% |
|  |  | 6. Roaming dogs are a problem in our neighbourhood. | CATI | Ordinal | 18.7 | 1 = strongly agree | 3.1% |
|  |  |  |  |  |  | 2 = agree | 11.7% |
|  |  |  |  |  |  | 3 = neither agree nor disagree | 4.1% |
|  |  |  |  |  |  | 4 = disagree | 55.0% |
|  |  |  |  |  |  | 5 = strongly disagree | 7.3% |
|  |  | 7. It’s a good place to buy a home. | CATI | Ordinal | 19.4 | 1 = strongly disagree | 1.2% |
|  |  |  |  |  |  | 2 = disagree | 5.5% |
|  |  |  |  |  |  | 3 = neither agree nor disagree | 3.5% |
|  |  |  |  |  |  | 4 = agree | 54.8% |
|  |  |  |  |  |  | 5 = strongly agree | 15.5% |
|  |  | 8. Bullying is a problem in our neighbourhood. | CATI | Ordinal | 22.7 | 1 = strongly agree | 1.9% |
|  |  |  |  |  |  | 2 = agree | 8.9% |
|  |  |  |  |  |  | 3 = neither agree nor disagree | 5.6% |
|  |  |  |  |  |  | 4 = disagree | 54.5% |
|  |  |  |  |  |  | 5 = strongly disagree | 6.4% |
|  |  | 9. There are a lot of families with young children living in our neighbourhood. | CATI | Ordinal | 20.4 | 1 = strongly disagree | 0.1% |
|  |  |  |  |  |  | 2 = disagree | 6.0% |
|  |  |  |  |  |  | 3 = neither agree nor disagree | 4.5% |
|  |  |  |  |  |  | 4 = agree | 54.8% |
|  |  |  |  |  |  | 5 = strongly agree | 14.2% |
| Neighbourhood cohesion | Neighbourhood social environment | Summed scores of 9 items: | CATI | Continuous | 36.7 | - | 33.8 ± 4.5 |
|  |  | 1. People are willing to help. | CATI | Ordinal | 22.3 | 1 = strongly disagree | 0.4% |
|  |  |  |  |  |  | 2 = disagree | 5.1% |
|  |  |  |  |  |  | 3 = neither agree nor disagree | 7.7% |
|  |  |  |  |  |  | 4 = agree | 55.3% |
|  |  |  |  |  |  | 5 = strongly agree | 9.2% |
|  |  | 2. Neighbours watch out for kids. | CATI | Ordinal | 22.9 | 1 = strongly disagree | 0.5% |
|  |  |  |  |  |  | 2 = disagree | 6.5% |
|  |  |  |  |  |  | 3 = neither agree nor disagree | 7.4% |
|  |  |  |  |  |  | 4 = agree | 53.5% |
|  |  |  |  |  |  | 5 = strongly agree | 9.1% |
|  |  | 3. It’s a close knit neighbourhood. | CATI | Ordinal | 20.8 | 1 = strongly disagree | 0.7% |
|  |  |  |  |  |  | 2 = disagree | 18.7% |
|  |  |  |  |  |  | 3 = neither agree nor disagree | 15.3% |
|  |  |  |  |  |  | 4 = agree | 39.2% |
|  |  |  |  |  |  | 5 = strongly agree | 5.3% |
|  |  | 4. I could borrow $10 from a neighbour. | CATI | Ordinal | 25.3 | 1 = strongly disagree | 2.3% |
|  |  |  |  |  |  | 2 = disagree | 20.4% |
|  |  |  |  |  |  | 3 = neither agree nor disagree | 5.1% |
|  |  |  |  |  |  | 4 = agree | 39.4% |
|  |  |  |  |  |  | 5 = strongly agree | 7.5% |
|  |  | 5. If there is a problem with neighbours, we can deal with it. | CATI | Ordinal | 21.7 | 1 = strongly disagree | 0.3% |
|  |  |  |  |  |  | 2 = disagree | 5.1% |
|  |  |  |  |  |  | 3 = neither agree nor disagree | 5.1% |
|  |  |  |  |  |  | 4 = agree | 62.0% |
|  |  |  |  |  |  | 5 = strongly agree | 5.9% |
|  |  | 6. The neighbours cannot be trusted. | CATI | Ordinal | 21.7 | 1 = strongly agree | 0.4% |
|  |  |  |  |  |  | 2 = agree | 6.6% |
|  |  |  |  |  |  | 3 = neither agree nor disagree | 6.0% |
|  |  |  |  |  |  | 4 = disagree | 54.2% |
|  |  |  |  |  |  | 5 = strongly disagree | 11.2% |
|  |  | 7. People will take advantage of you. | CATI | Ordinal | 22.4 | 1 = strongly agree | 0.9% |
|  |  |  |  |  |  | 2 = agree | 6.7% |
|  |  |  |  |  |  | 3 = neither agree nor disagree | 5.3% |
|  |  |  |  |  |  | 4 = disagree | 55.6% |
|  |  |  |  |  |  | 5 = strongly disagree | 9.0% |
|  |  | 8. People you don’t know will greet you or say hello to you. | CATI | Ordinal | 19.2 | 1 = strongly disagree | 0.3% |
|  |  |  |  |  |  | 2 = disagree | 6.6% |
|  |  |  |  |  |  | 3 = neither agree nor disagree | 5.9% |
|  |  |  |  |  |  | 4 = agree | 61.4% |
|  |  |  |  |  |  | 5 = strongly agree | 6.6% |
|  |  | 9. People of different backgrounds don’t talk to each other. | CATI | Ordinal | 21.7 | 1 = strongly agree | 0.9% |
|  |  |  |  |  |  | 2 = agree | 17.0% |
|  |  |  |  |  |  | 3 = neither agree nor disagree | 7.6% |
|  |  |  |  |  |  | 4 = disagree | 49.3% |
|  |  |  |  |  |  | 5 = strongly disagree | 3.6% |
| Neighbourhood connection | Neighbourhood social environment | Summed scores of 5 items: | CATI | Continuous | 35.9 | - | 18.6 ± 3.2 |
|  |  | 1. Parents in this neighbourhood know their children’s friends. | CATI | Ordinal | 24.8 | 1 = strongly disagree | 0.4% |
|  |  |  |  |  |  | 2 = disagree | 7.6% |
|  |  |  |  |  |  | 3 = neither agree nor disagree | 6.1% |
|  |  |  |  |  |  | 4 = agree | 53.7% |
|  |  |  |  |  |  | 5 = strongly agree | 7.4% |
|  |  | 2. Adults in this neighbourhood know who the local children are. | CATI | Ordinal | 25.5 | 1 = strongly disagree | 0.7% |
|  |  |  |  |  |  | 2 = disagree | 10.3% |
|  |  |  |  |  |  | 3 = neither agree nor disagree | 10.6% |
|  |  |  |  |  |  | 4 = agree | 48.2% |
|  |  |  |  |  |  | 5 = strongly agree | 4.6% |
|  |  | 3. There are adults in this neighbourhood that the children can look up to. | CATI | Ordinal | 28.3 | 1 = strongly disagree | 0.5% |
|  |  |  |  |  |  | 2 = disagree | 9.3% |
|  |  |  |  |  |  | 3 = neither agree nor disagree | 10.0% |
|  |  |  |  |  |  | 4 = agree | 46.6% |
|  |  |  |  |  |  | 5 = strongly agree | 5.3% |
|  |  | 4. Parents in this neighbourhood generally know each other. | CATI | Ordinal | 22.6 | 1 = strongly disagree | 0.5% |
|  |  |  |  |  |  | 2 = disagree | 12.5% |
|  |  |  |  |  |  | 3 = neither agree nor disagree | 10.9% |
|  |  |  |  |  |  | 4 = agree | 48.2% |
|  |  |  |  |  |  | 5 = strongly agree | 5.3% |
|  |  | 5. You can count on adults in this neighbourhood to watch out that children are safe and don’t get in trouble. | CATI | Ordinal | 24.9 | 1 = strongly disagree | 0.5% |
|  |  |  |  |  |  | 2 = disagree | 8.1% |
|  |  |  |  |  |  | 3 = neither agree nor disagree | 10.2% |
|  |  |  |  |  |  | 4 = agree | 50.3% |
|  |  |  |  |  |  | 5 = strongly agree | 6.0% |
| *Built environment* | | | | | | | |
| Distance to school | - | Distance to school (in metres) along softGIS school routes | GIS | Continuous | 11.5 | - | 2783.7 ± 3557.7 |
|  |  | Distance to school (log-transformed) along softGIS school routes | GIS | Continuous | 11.5 | - | 7.4 ± 1.0 |
| Residential density | Active mobility environment | Ratio of residential dwellings to the residential land area (i.e., without water) of 80 m softGIS route buffer | GIS | Continuous | 11.6 | - | 28.8 ± 10.8 |
| Street connectivity | Active mobility environment | Ratio of number of intersections with three or more intersecting streets to the land area of 80 m softGIS route buffer | GIS | Continuous | 11.6 | - | 56.6 ± 19.2 |
| High traffic exposure | Active mobility environment | Length of high traffic roads within 80 m softGIS route buffer weighted by inverse softGIS route distance | GIS | Continuous | 11.6 | - | 5.9 ± 4.9 |
| Low traffic exposure | Active mobility environment | Length of low traffic roads within 80 m softGIS route buffer weighted by inverse softGIS route distance | GIS | Continuous | 11.6 | - | 10.5 ± 8.2 |
| CATI = computer-assisted telephone interviewing. GIS = geographic information systems. *Dummy variable. †Frequencies (%) for binary or ordinal variables; mean ± standard deviation for continuous variables. | | | | | | | |
