# Supplementary figures and images for: Assessment of direct and indirect associations between children active school travel and environmental, household and child factors using structural equation modelling
Source: Int J Behav Nutr Phys Act. 2019 Apr 5;16:32. doi: 10.1186/s12966-019-0794-5 (PMC6451289; doi:10.1186/s12966-019-0794-5)

# Additional file 4

The hypothesised full structural equation modelling


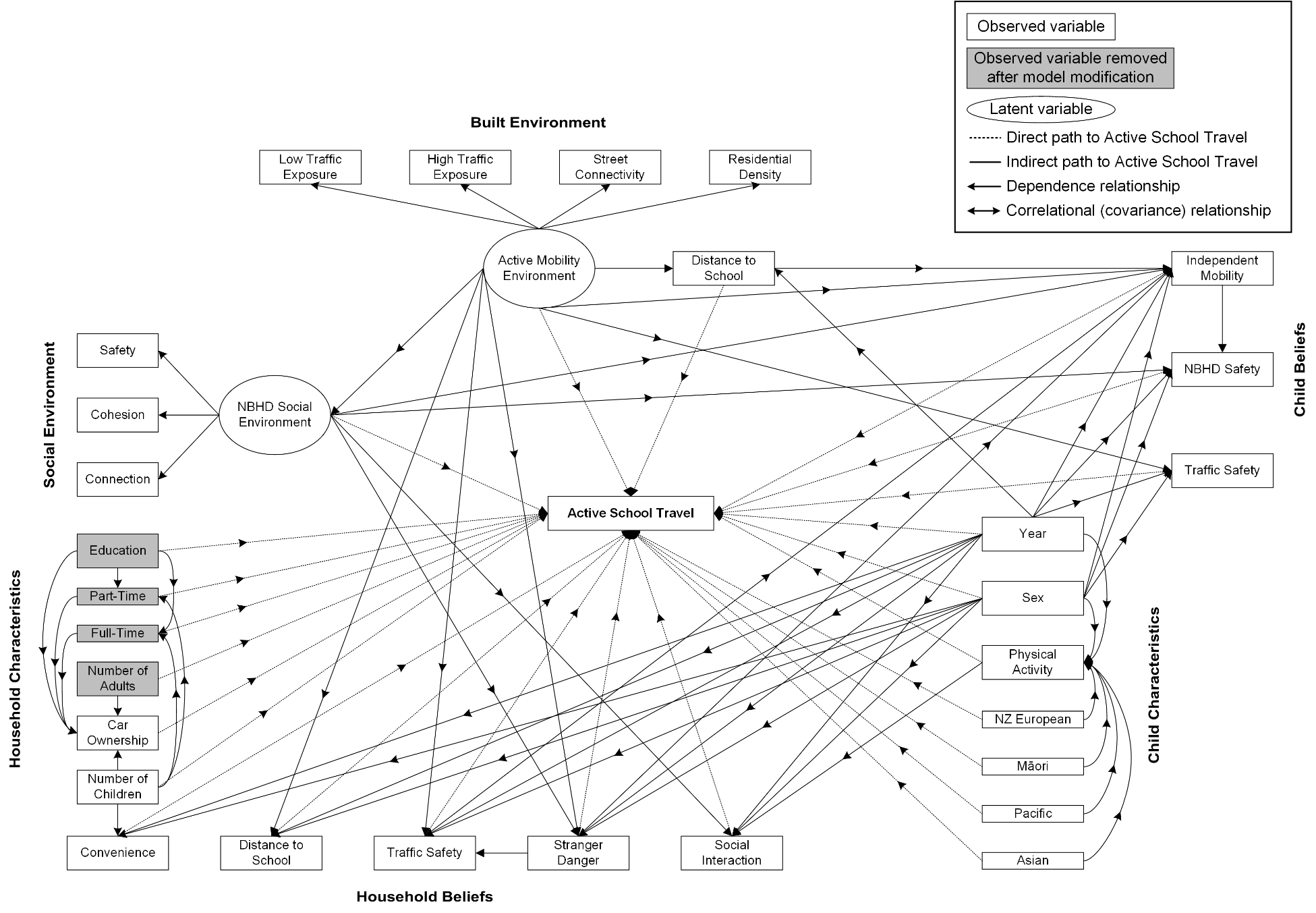

Supplement: Supplementary file 4 — The hypothesised full structural equation modelling. (DOCX 561 kb) [file 12966_2019_794_MOESM4_ESM.docx]
