# Supplementary material for: Assessment of direct and indirect associations between children active school travel and environmental, household and child factors using structural equation modelling
Source: Int J Behav Nutr Phys Act. 2019 Apr 5;16:32. doi: 10.1186/s12966-019-0794-5 (PMC6451289; doi:10.1186/s12966-019-0794-5)

# Additional file 5

Unstandardised estimated coefficients of the structural equation model of children’s active travel to school. Root mean square error of approximation (RMSEA) = 0.04, comparative fit index (CFI) = 0.94, Tucker-Lewis index (TLI) = 0.92.


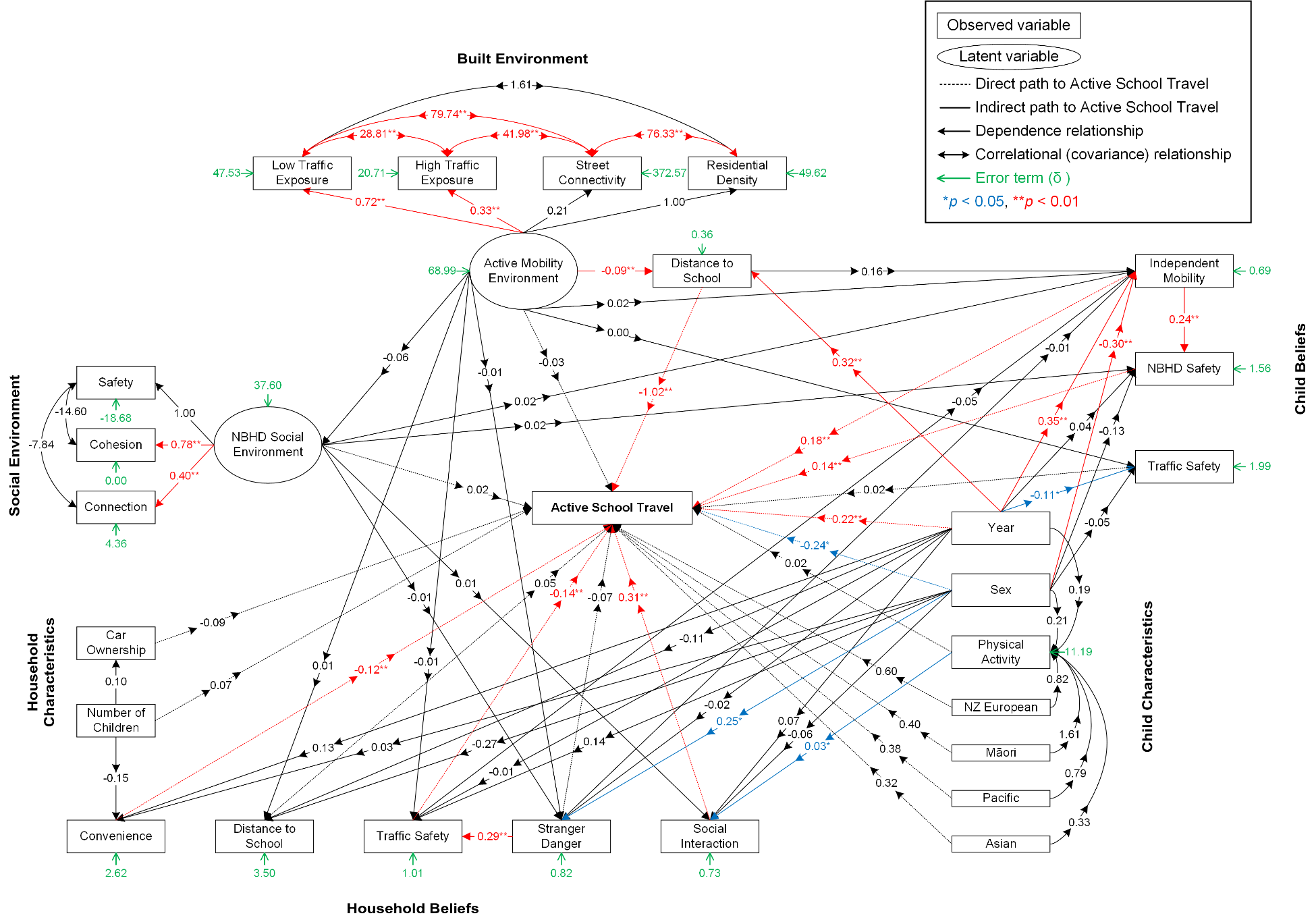

Supplement: Supplementary file 5 — Unstandardised estimated coefficients of the structural equation model of children’s active travel to school. Root mean square error of approximation (RMSEA) = 0.04, comparative fit index (CFI) = 0.94, Tucker-Lewis index (TLI) = 0.92. (DOCX 606 kb) [file 12966_2019_794_MOESM5_ESM.docx]
